# Novel NSAID Analogs Exhibit Anti-Leukemic Activity Through Modulation of Apoptotic and Survival Pathways

**DOI:** 10.3390/ijms27093850

**Published:** 2026-04-26

**Authors:** Hind A. Alkhatabi, Mohammed Basabrain, Alaa G. Alahmadi, Shiekhah M. Alzahrani, Yosra A. Muhammad, Maha Almuhaiyawi, Maha M. Alreemi, Reem M. Alotibi, Roaa M. Alreemi, Heba A. Alkhattabi, Reem N. Hassan, Wedad M. Al-Bishri, Mohammed El-Mezgueldi, Abdelsattar M. Omar

**Affiliations:** 1 Department of Biological Sciences, College of Science, University of Jeddah, Jeddah 21956, Saudi Arabia; 2 Institute of Genomic Medicine Sciences, Faculty of Applied Medical Sciences, King Abdulaziz University, Jeddah 21589, Saudi Arabia; 3 Department of Pharmaceutical Chemistry, Faculty of Pharmacy, King Abdulaziz University, Jeddah 21589, Saudi Arabia; 4 King Fahd Medical Research Center, King Abdulaziz University, P.O. Box 80216, Jeddah 21589, Saudi Arabia; 5 Collage of Medicine, King Saud bin Abdulaziz University for Health Sciences, Jeddah 22384, Saudi Arabia; 6 Department of Biological Sciences, Faculty of Sciences, King Abdulaziz University, Jeddah 21589, Saudi Arabia; 7 Randall Centre for Cell & Molecular Biophysics, King’s College London, New Hunt’s House, Room3/28a, London SE1 1UL, UK; 8 College of Health and Life Sciences, School of Biosciences, University of Aston, Birmingham B4 7ET, UK

**Keywords:** AML, NSAID derivatives, apoptosis, mitochondrial dysfunction, cell cycle arrest, antioxidant, ROS

## Abstract

Acute myeloid leukemia (AML) is a complex blood cancer that primarily affects relapsing or refractory patients receiving conventional chemotherapy. Nonsteroidal anti-inflammatory drugs (NSAIDs) have anticancer properties with restricted clinical efficacy attributable to cyclooxygenase (COX)-induced toxicities. To address this issue, a group of benzylamide analogs of the classical NSAIDs (NSI-1–NSI-9) were developed and synthesized to mask the carboxylic acid moiety and minimize COX-induced adverse effects while maintaining anticancer activity. The cytotoxic effect of such substances has been demonstrated in some leukemia cell lines (HL-60, MV4-11, KG1a, and K562). NSI-5 exerted the highest anti-leukemic activity among these sulindac analogs, as determined at a sub-micromolar level in all cell lines studied, by IC50. This mechanistic data also demonstrated that NSI-5 induced apoptosis that was dose-dependent, especially in HL-60 cell lines, and increased the sub-G1 cell fraction. This apoptotic process was also accompanied by a significant decrease in mitochondrial membrane potential, which is characteristic of the induction of the intrinsic apoptotic process. Interestingly, NSI-5 decreased the intracellular reactive oxygen species (ROS) and the expression of most antioxidants (catalase and glutathione synthetase), as well as the redox balance. Gene characterization in vitro also suggested activation of apoptotic pathways, where expression of *Bax*, *Bak1*, and *Caspase-3* increased, suggesting a potential p53-independent apoptotic pathway, in contrast to control for *Bcl-2* expression. Collectively, these findings indicate that NSI-5 is a promising in vitro anti-leukemic lead compound, with activity associated with mitochondrial dysfunction and altered redox regulation. The observed effects are consistent with previously reported COX-independent activity of structurally related NSAID derivatives, and support further investigation of NSI-5 in preclinical models.

## 1. Introduction

Acute myeloid leukemia (AML) is a rapidly progressing hematologic malignancy characterized by the accumulation of immature myeloid blasts in the bone marrow. It is the most prevalent acute leukemia in adults and is associated with poor prognosis, particularly in older patients (and other medically unfit patients) despite treatment advances [1]. High recurrence rates and low tolerability of intensive therapy indicate the urgent need for safer treatment interventions. Long-lasting inflammatory processes contribute to leukemogenesis and poor disease-related outcomes. The elevated level of pro-inflammatory cytokines in the bone marrow niche has also correlated with better leukemic stem cell survival and resistance to treatment, as high levels of pro-inflammatory cytokines are known [2], which implies inflammatory pathway targeting as an important therapeutic approach in this area. Epidemiologic and experimental evidence also suggest NSAIDs and anti-cancer agents (aspirin included) are considered anti-cancer drugs; occasional use of the drug shows some decreased risk of leukemia [3].

NSAIDs have also shown unique anti-tumor effects that are superior to chemoprevention effects as indicated by the suppression of proliferation and stimulation of apoptosis in solid tumors and hematologic tumors [4,5]. Especially, COX inhibition and inhibition of prostaglandin E_2_ signaling, which is a driving factor for tumor growth and immune escape, are involved [4,5,6]. Inhibition of COX can be involved in certain NSAID activities. Agents including R-etodolac (SDX-101) and a closely related analog (SDX-308 and SDX-309) have demonstrated cytotoxic and pro-apoptotic activity in leukemia models, and other derivatives have moved into clinical studies [7]. Conventional NSAIDs, however, are not widely available clinically because of potential impacts on the gastrointestinal, renal, and cardiovascular tract due to potential long-term or high-dose toxicity [8,9]. Thus, no NSAID is drug-approved for cancer therapy. Current strategies involve using NSAIDs with other drugs and developing structurally modified derivatives for use as anticancer agents with lower COX-induced toxic effects. Various new-generation NSAID scaffolds have demonstrated enhanced safety and anticancer efficacy in preclinical studies and may be useful for future studies targeting NSAIDs to produce anticancer drug leads [6,9,10].

In view of these insights, we addressed the problem of developing and testing new analogs of NSAIDs to maximize their anti-leukemic activity. Our first approach was to convert the carboxylic acid group, which is indispensable for COX binding and GI toxicity, found in most NSAIDs, into an N-benzyl amide function. Theoretically, we anticipated that these structural changes would (a) decrease COX-1/2-inhibitory activity (and thereby perhaps diminish any typical NSAID side effects) and (b) potentially provide molecules with novel cytotoxic properties toward leukemia cells.

## 2. Results

### 2.1. Synthesis and Characterization of NSAID-Benzylamide Derivatives

**Figure 1:**
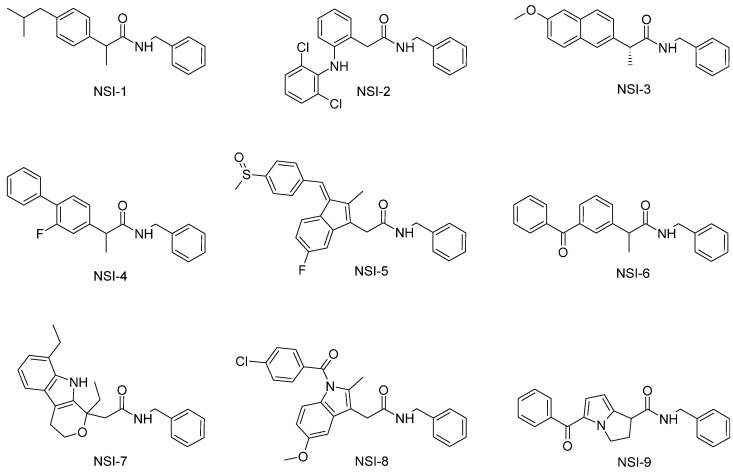
Chemical structures of benzylamine derivatives of ibuprofen (NSI-1), diclofenac (NSI-2), naproxen (NSI-3), flurbiprofen (NSI-4), sulindac (NSI-5), ketoprofen (NSI-6), etodolac (NSI-7), indomethacin (NSI-8), and ketorolac (NSI-9).

**Scheme 1:**
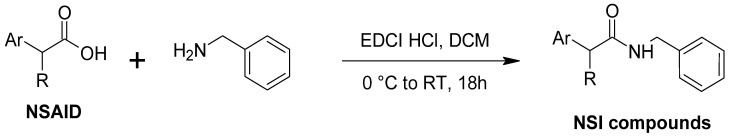
General synthesis method for NSI derivatives.

The molecular structures of the synthetic NSAID-benzylamide analogs are shown in Figure 1. All nine derivatives (NSI-1–NSI-9) were obtained via EDCI-mediated coupling of parent NSAID carboxylic acids with benzylamine under mild conditions (Scheme 1). The reactions were initiated at 0 °C and stirred overnight at room temperature to obtain the target amides in low- to moderate isolated yields after chromatographic purification. The identities were confirmed by 1H/13C NMR and LC-MS, respectively, and the analytical characteristics of all compounds indicated reasonable purity.

Typical spectral and analytical data showed the conversion of parent NSAID carboxylic acid to benzylamide derivatives (NSI-1–NSI-9). The carboxylic OH signal was absent from all compounds; however, we observed a new amide NH resonance at 8.1–8.9 ppm and a downfield shift in the benzylic methylene protons (≈4.1–4.4 ppm). LC–MS analysis showed distinct [M+H]+ that were consistent with calculated molecular masses. The melting points indicated that the material was solid or semi-solid, consistent with NSAID-derived scaffolds, and chromatographic profiles were also available. Collectively, these data demonstrate a synthetic and structural realization of a NSI library before biological analyses.

### 2.2. Biological Activity

#### 2.2.1. In Vitro Cytotoxic Evaluation of Novel NSAID–Benzylamide Analogs Against Leukemic Cell Lines

**Figure 2:**
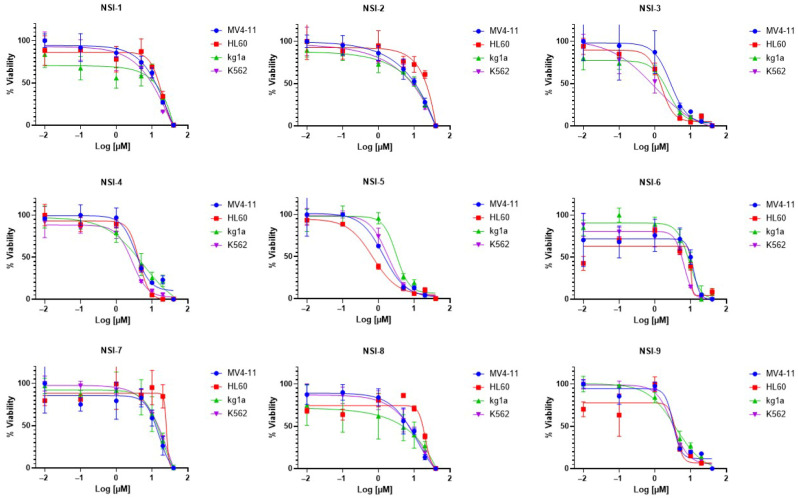
Dose–response curves of compounds in leukemia cell lines. Cells were treated with increasing concentrations of the compounds for 48 h, and cell viability was measured using the CellTiter^®^-Blue Cell Viability Assay. Dose–response curves were generated using nonlinear regression with a four-parameter variable-slope model from three independent experiments (n = 3).

Selection of NSI compounds was conducted for the analysis of cytotoxicity of nine NSI compounds in HL-60, MV4-11, KG1a, and K562 leukemia cell lines by the Celltiter-Blue viability assay. The results are shown as dose–response curves in Figure 2 and IC_50_ values in Table 1. Among the screened products, NSI-3, NSI-4, NSI-5, NSI-6, and NSI-9 were found to be the most cytotoxic among all the evaluated leukemia cell lines, and NSI-5 was the most potent overall. The IC_50_ values of NSI-3, NSI-4, NSI-6 and NSI-9 fell between 1 and 11.80 μM and NSI-5 showed the minimum IC_50_ from all four cell lines (0.60 to 1.50 μM). In contrast, NSI-1, NSI-2, NSI-7 and NSI-8 showed the weakest cytotoxic effects (IC_50_ 12.20 to >50 μM) and NSI-2 exhibited minimal activity with IC_50_ greater than 50 μM in all studied cell lines. NSI-5 was recognized as the most active compound with HL-60 displaying an IC_50_ of 0.61 μM, being the most sensitive cell line.

Given its consistently superior potency across all leukemia models tested, NSI-5 was selected as the lead compound for subsequent investigations. Although several other derivatives (e.g., NSI-3, NSI-4, and NSI-9) also demonstrated measurable cytotoxic activity, their potency was comparatively lower and less consistent across the tested cell lines. While NSI-5 demonstrated the highest potency across the evaluated leukemia cell lines, a structural comparison across the series provides valuable structure–activity relationship (SAR) insights. The superior activity of NSI-5 can plausibly be attributed to the unique spatial geometry conferred by its indene core and the exocyclic benzylidene double bond, combined with the distinct electronics of its *para*-methylsulfinyl group. Compared to less active derivatives such as NSI-2 (which possesses a highly flexible diphenylamine scaffold) or NSI-4 (a freely rotating biphenyl system), the rigid conjugated system of NSI-5 restricts its distal phenyl ring to a specific conformational space. Furthermore, while compounds like NSI-2, NSI-4, and NSI-8 rely primarily on lipophilic halogen substitutions at their extremities, the methylsulfinyl moiety of NSI-5 introduces a localized polar region capable of acting as a hydrogen bond acceptor. This unique combination of conformational rigidity and an amphiphilic electronic profile likely facilitates an optimal fit and enhanced target engagement, driving its pronounced anti-leukemic effect relative to the other analogs in the series.

#### 2.2.2. NSI-5 Analogs Induced Apoptosis in Multiple Leukemic Cell Lines in a Dose-Dependent Manner

**Figure 3:**
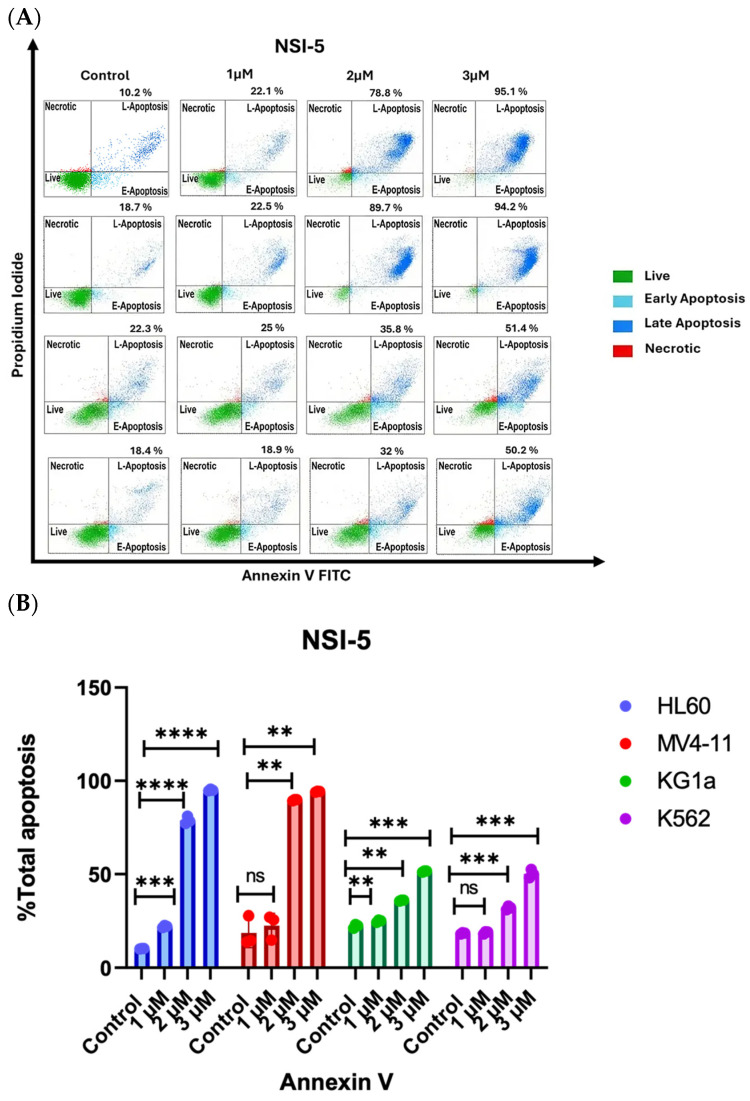
Detection of total apoptosis in leukemic cell lines by NSI-5 treatment. (**A**) Representative flow cytometric scatter plots of HL-60, MV4-11, KG1a, and K562 cells after 48 h of treatment with NSI-5 (1, 2, and 3 μM) and staining with Annexin V-FITC/PI. (**B**) The mean percentage of total apoptotic cells is plotted from three independent experiments (n = 3), with error bars representing ± SEM. *p*-values were determined using two-way ANOVA in GraphPad Prism 10.1.1 software. ns = non-significant value, ** *p* ≤ 0.01, *** *p* ≤ 0.001, and **** *p* ≤ 0.0001.

To evaluate whether the cytotoxic effect of NSI-5 was mediated by programmed cell death, the apoptotic status of four leukemic cell lines (HL-60, MV4-11, KG-1a, and K562) was detected using Annexin V-FITC/PI staining. We previously reported that NSI-5 induces apoptosis in a dose-dependent and cell-type-specific manner, which is in line with the results of the first cell viability assays (Figure 3A). The HL-60 cell line exhibited the highest sensitivity to treatment; total apoptosis increased from 10.20% in control cells to 22.16%, 78.86%, and 95.06% at concentrations of 1, 2, and 3 μM, respectively. Conversely, although the 1 μM dose did not induce apoptosis in MV4-11 and K562 cells, higher doses (2 and 3 μM) induced strong apoptotic reactions. Specifically, total apoptosis in MV4-11 cells increased to 89.70% and 94.20% relative to the control (18.73%), whereas K562 cells showed a 32.03% and 50.20% increase relative to the control (18.43%) at these concentrations. The KG-1a cell line exhibited a modest dose dependency, with apoptosis levels of 25%, 35.86%, and 51.46% relative to the control (22.30%) (Figure 3B). These data confirm that NSI-5 exerts its anti-leukemic activity primarily through the induction of apoptosis, with different potencies across different myeloid leukemia models.

#### 2.2.3. NSI-5 Compound Arrested the Cell Cycle at the Sub-G1 Phase of Leukemic Cell Lines

**Figure 4:**
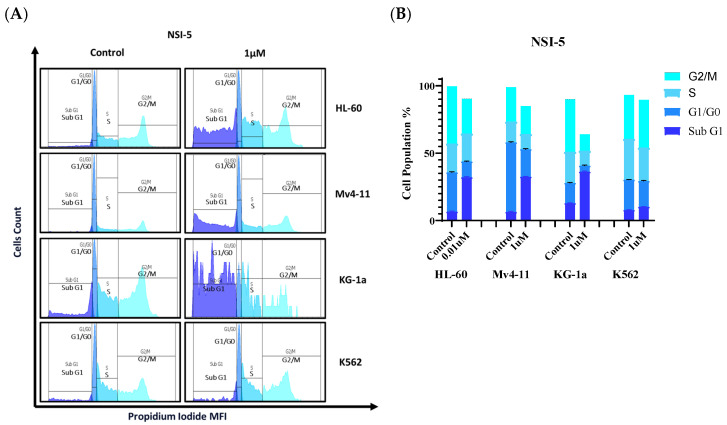
Distribution of the cell cycle phases in leukemic cell lines after NSI-5 treatment using PI dye and flow cytometry. (**A**) Representative flow cytometric plots of the cell cycle phase distribution in leukemic cells. Cells were treated with 1 µM NSI-5 for 48 h and stained with Propidium Iodide MFI for analysis by flow cytometry. (**B**) Representative bar graphs showing the percentage of cells in different phases of the cell cycle, presented as the mean ± standard error of the mean (SEM) from two independent experiments (n = 2). Statistical differences between groups were analyzed using two-way analysis of variance (ANOVA) in GraphPad Prism version 10.1.1.

NSI-5 affected cell cycle progression, as assessed by flow cytometry analysis of cells in different phases from propidium iodide (PI) staining in HL-60, MV4-11, KG-1a, and K562 cell lines. Treatment with 1 μM NSI-5 caused high accumulation of cells in the sub-G1 phase (as shown in Figure 4), indicative of DNA fragmentation and the onset of apoptosis in all tested cell lines, but showed no major changes in other cell cycle phases. The maximal enrichment of the sub-G1 population occurred in HL-60 (31.60% vs. 6.10% in the control), MV4-11 (31.90% vs. 5.90% in the control), and KG-1a (37.7% vs. 12.3% in the control) cells. In contrast, the change in K562 cells was not immediately visible, with a sub-G1 level of 9.8% compared to 7.3% in the untreated cells. Mild increases in the sub-G1 fraction at 1 μM in K562 cells are consistent with annexin V-FITC/PI staining, because the same concentration induced marginal apoptosis in this cell line. Accordingly, these findings provide evidence for the role of apoptosis in NSI-5-induced cytotoxicity.

#### 2.2.4. NSI-5 Compound Altered Mitochondrial Membrane Potential (MMP) in Leukemic Cell Lines

**Figure 5:**
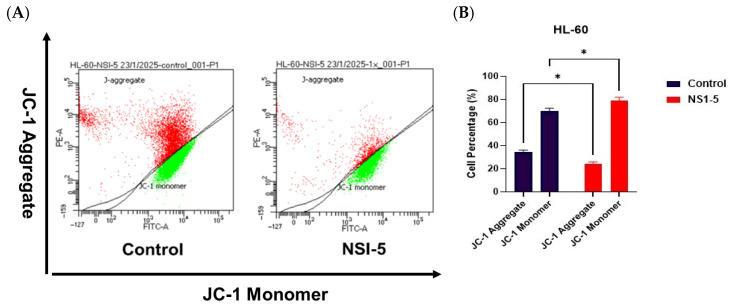
NSI-5 induces depolarization of the mitochondrial membrane in HL-60 cells. (**A**) Flow cytometry plots showing MMP variation in HL-60 cells after NSI-5 treatment. The shift from red (JC-1 aggregates) to green (JC-1 monomers) fluorescence indicates mitochondrial depolarization. (**B**) The bar chart presents a quantitative analysis of changes in MMP, and bars illustrate the average percentages of JC-1 aggregates and monomers in control and NSI-5-treated HL-60 cells. The data are shown as mean ± SEM calculated from at least three independent experiments (n = 3). Statistical significance was determined using a two-way ANOVA followed by Tukey’s post hoc analysis (*p* ≤ 0.05). Significance is stated as * *p* ≤ 0.05.

We examined mitochondrial membrane potential (ΔΨm) in HL-60 cells using JC-1 fluorochrome to assess NSI-5-mediated apoptosis. The shift from red JC-1 aggregates to green JC-1 monomers indicates mitochondrial depolarization and dysfunction. A remarkable change is best seen in Figure 5, where the histogram clearly shows the degree of mitochondrial dysfunction in HL-60 cells, resulting in a monomer count of 79.16% versus 69.85% in the control group, with aggregate fractions dropping to 24.43%. Therefore, the increased JC-1 monomers in HL-60 cells confirmed mitochondrial membrane depolarization. NSI-5 was shown to induce mitochondrial dysfunction, which is required in the intrinsic apoptotic pathway by the principal mechanism of its cytotoxic effect.

#### 2.2.5. NSI-5 Treatment Suppresses Intracellular ROS Generation in HL-60 Cells

**Figure 6:**
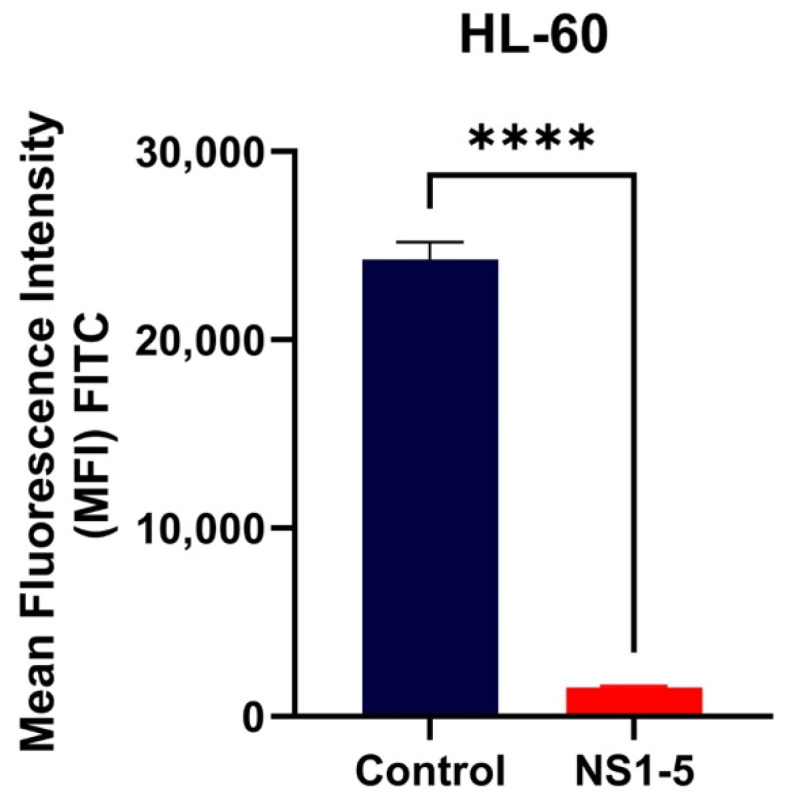
Measurement of intracellular ROS levels in HL-60 cells after NSI-5 treatment for 48 h. The plots present the values of MFI for the intracellular ROS dye (FITC) in the control and NSI-5 groups. Comparisons of MFI between groups were made using an unpaired *t*-test in GraphPad Prism 10.1.1 software. The represented values are expressed as the mean of three independent experiments ± standard error of the mean (n = 3 ± SEM). **** *p* ≤ 0.0001.

To confirm the linkage between NSI-5-induced apoptosis and oxidative stress, we measured intracellular ROS levels of HL-60 cells via ROS-sensitive fluorogenic probe DCFDA and flow cytometry. Interestingly, even though mitochondrial depolarization and apoptosis were induced through NSI-5 induction, ROS levels were still remarkably decreased in response to NSI-5 treatment. The mean fluorescence intensity (MFI) of the ROS-sensitive probe in HL-60 cells is shown in Figure 6 and decreased from 24,269 in untreated controls to 1538 in NSI-5 (1 μM) treatment (**** *p* < 0.0001). The reduction in MFI suggests that NSI-5 can attenuate basal ROS production or is efficient in scavenging intracellular radicals. This is significant when examining the concomitant collapse of mitochondrial membrane potential (ΔΨm) in these cells (Figure 6). Given this background, it seems that the cytotoxic activity of NSI-5 is not dependent on oxidative damage triggered by ROS, suggesting that NSI-5 might disrupt redox regulation in cells or induce the mitochondrial respiratory chain to a point where ROS production is stopped, while activating the intrinsic apoptotic pathway.

#### 2.2.6. NSI-5 Orchestrates p53-Independent Apoptosis via Mitochondrial Collapse and Antioxidant Depletion in HL-60 Cells

**Figure 7:**
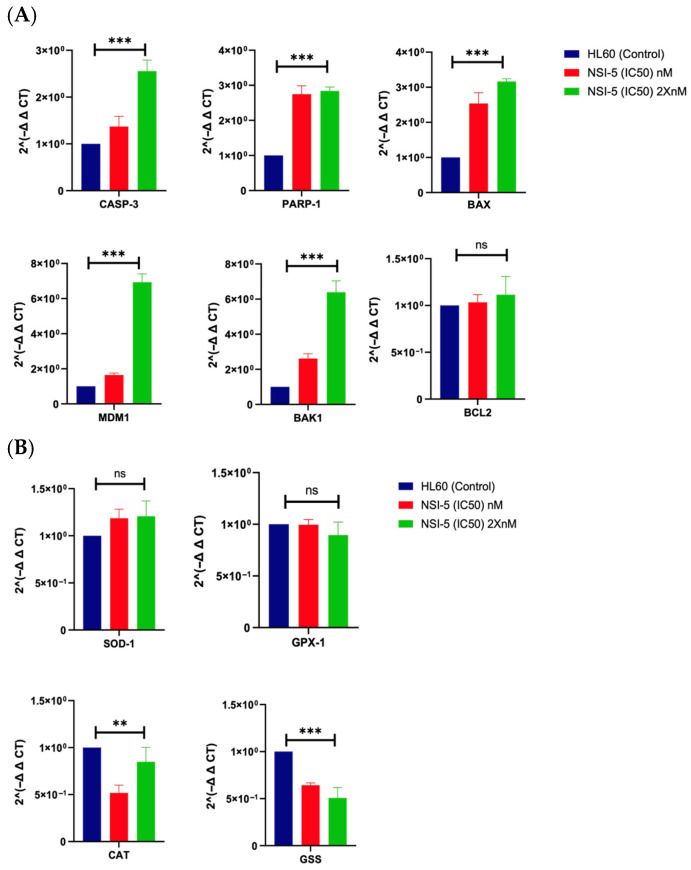
NSI-5 exposure for 48 h alters the expression of apoptosis-related genes and antioxidant markers in HL-60 cells. Gene expression levels were determined by quantitative real-time PCR and normalized to the internal housekeeping gene (GAPDH). HL-60 cells were treated with NSI-5 at 1 and 2 μM and compared with the untreated control. Data are presented as the standard error of the mean (SEM) from at least three independent experiments (n = 3). Statistical significance was analyzed using one-way ANOVA, followed by a post hoc multiple-comparison test (** *p* ≤ 0.01, *** *p* ≤ 0.001). (**A**) Level of mRNA expression of apoptosis-related genes in HL-60 cells. (**B**) Level of mRNA expression of antioxidant-related genes in HL-60 cells. Blue bars represent control (untreated), whereas the red and green bars represent cells treated with 1 and 2 μM NSI-5, respectively. ns: non-significant.

To better understand how NSI-5 induces programmed cell death at the molecular level, we quantified the RNA levels of key regulators in the apoptotic signal transduction pathways. HL-60 cells were treated with 1 and 2 μM NSI-5 for 48 h, and transcriptional level changes were evaluated using RT-qPCR. The findings in Figure 7A show a significant increase in the expression of several critical pro-apoptotic stimulants. For 1 μM NSI-5 treatment, there was a significant increase in the pore-forming *Bcl-2* family members (*Bax* and *Bak1*) and executioner protease caspase-3 (*** *p* ≤ 0.001). In addition, *PARP-1* and *MDM1* levels increased. Interestingly, the pro-apoptotic transcripts were significantly induced in host cells, whereas the expression of the anti-apoptotic gene *Bcl-2* remained constant compared to control cells (*p* > 0.05). This hyper-specific expression re-arranged the intracellular balance in favor of the pro-apoptotic state, as it upregulated the *Bax*/*Bcl-2* ratio, which is a well-established factor in susceptibility to apoptosis. The same pattern of expression persisted at 2 μM concentration. Such molecular observations confirm our previous observations of mitochondrial membrane depolarization and sub-G1 phase accumulation, and highlight that NSI-5 activates the intrinsic mitochondrial apoptotic cascading pathway to facilitate leukemic cell death.

Moreover, we assessed mRNA expression of the predominant endogenous antioxidant enzymes *SOD-1*, *CAT*, *GPX-1*, and *GSS* after NSI-5 treatment to clarify the association between the observed reduction in intracellular ROS and the cellular antioxidant machinery. According to Figure 7B, treatment with 1 and 2 μM of NSI-5 produced significant and dose-dependent downregulation of *Catalase* (*CAT*) and *Glutathione Synthetase* (*GSS*) mRNA levels (** *p* ≤ 0.01 and *** *p* ≤ 0.001, respectively). In contrast, the expression of SOD-1 and GPX-1 was not significantly affected (*p* > 0.05). These molecular alterations help illuminate the previously mentioned fall in ROS mean fluorescence intensity (Figure 6). The low levels produced by *GSS*, an essential enzyme in the synthesis of glutathione, and *CAT* in particular, indicate that NSI-5 impairs the capacity of the cell to maintain oxidative homeostasis. Although ROS bursts are induced by many pro-apoptotic molecules, the sharp reduction in ROS levels in this study, along with the destruction of antioxidant transcripts, indicates that NSI-5 induces a redox imbalance or induces cell-mediated collapse of the mitochondrial respiratory chain, where the buffering capacity of antioxidant proteins may be lost. In summary, exhaustion of the antioxidant system, namely, the glutathione pathway, probably sensitizes HL-60 cells to the intrinsic apoptotic signaling previously elucidated, resulting in efficient leukemic cell death.

## 3. Discussion

Classical NSAIDs have recently been re-imagined by transforming the carboxyl side into a benzylamide. This carboxylate is thought to allow COX binding, thus serving as a restricting factor in long-term NSAID use for gastrointestinal and cardiovascular toxicity [11,12], and the suppression of this acidic site could reduce COX activity and facilitate interaction with other anticancer targets [11,13]. Consistent with this rationale, multiple studies have demonstrated that non-acidic NSAID derivatives lacking the carboxylate group retain significant anticancer activity despite markedly reduced or absent COX inhibitory function [11,13,14,15]. Mild amide-coupling assays yielded few benzylamide derivatives, with sensitive functions (halogens, heteroaromatics, and sulfoxides) retained. Spectroscopic analysis of the former showed a clean amide appearance for a variety of NSAID backbones, indicative of this remodeling method.

These results are supported by prior reports showing that non-acidic NSAID analogs maintain or enhance anticancer activity while exhibiting minimal COX inhibition, supporting COX-independent mechanisms described in the literature [11,13]. Removing the carboxyl group often diminishes COX-inhibition and has been associated with the activation of alternative anticancer pathways rather than prostaglandin suppression; sulindac amides and sulindac sulfone cause apoptosis via cGMP- and Wnt/β-catenin-related pathways and drive poor COX activity [16,17]. The celecoxib-derivatives 2,5-dimethyl-celecoxib and OSU-03012 also exhibit excellent cytotoxicity, in addition to COX-2, due to endoplasmic-reticulum stress and mitochondrial apoptosis [14].

Hence, the NSI series was constructed as a systematic approach to prepare the carboxyl functional group of a few NSAIDs into benzylamides using a relatively conserved scaffold. Of the synthetic derivatives, sulindac benzylamide (NSI-5) was selected as the rational lead. Among the synthesized derivatives, NSI-5 consistently exhibited the highest anti-leukemic activity across all tested cell lines and was therefore selected for further mechanistic investigation. While several analogs (e.g., NSI-3, NSI-4, and NSI-9) demonstrated measurable cytotoxicity, their effects were less potent and less consistent. The enhanced activity of NSI-5 may be attributed to its distinct structural features. In particular, the indene core and exocyclic benzylidene double bond impose a rigid, conjugated framework that likely restricts conformational flexibility compared to more flexible analogues such as NSI-2 and NSI-4. This structural rigidity may facilitate more favorable interactions with cellular targets. In addition, the presence of a para-methylsulfinyl group introduces a localized polar region capable of hydrogen bond acceptance, distinguishing NSI-5 from derivatives primarily bearing lipophilic substitutions. This combination of conformational constraint and amphiphilic electronic properties may contribute to enhanced target engagement and biological activity, and similar to the previous statements, sulindac amide analogs are anticancer-active molecules with weaker COX binding in many cancer cases and also in acute lymphoblastic leukemia cells [14,15,18]. However, direct COX enzymatic activity was not measured in the present study, and therefore COX-independence is inferred from structural rationale and prior literature and remains to be explored in future studies.

In conclusion, owing to its potent antiproliferative properties, IC50 between 0.60 and 1.50 μM was shown to exhibit strong cytotoxicity in NSI-5, making it the most potent analog of NSI in all selected leukemia cell lines. Annexin V-FITC/PI staining also confirmed that NSI-5 induced apoptotic effects, with HL-60 cells showing the maximum apoptotic effect, indicating that NSI-5-dependent cytotoxic actions can be accounted for by its inherent characteristics. In line with these findings confirming apoptotic DNA fragmentation, general cell cycle distribution analysis revealed an accumulation of cell assemblages in the sub-G1 phase, especially in HL-60 cells, supporting apoptotic-mediated DNA fragmentation. HL-60 cells were studied to further elucidate an underlying pathway based on redox and mitochondrial reactions. NSI-5 significantly reduced basal intracellular ROS levels despite inducing marked mitochondrial membrane depolarization. Rather than indicating a direct antioxidant or radical-scavenging effect, this reduction in ROS may reflect impaired mitochondrial electron transport activity following the collapse of ΔΨm, as mitochondria are a major source of intracellular ROS generation. Disruption of mitochondrial function can diminish electron leakage from the respiratory chain, thereby lowering measurable ROS levels as a secondary consequence of metabolic dysfunction. Importantly, this decrease in ROS occurred concurrently with significant downregulation of key antioxidant genes, particularly *CAT* and *GSS*, arguing against enhanced antioxidant capacity as the underlying mechanism. Instead, these findings suggest a state of redox imbalance characterized by compromised redox buffering and mitochondrial dysfunction. This altered redox and metabolic state likely contributes to the activation of intrinsic apoptotic signaling, as evidenced by the upregulation of *Bax*, *Bak1*, *caspase-3*, *PARP-1*, and *MDM1*, while *Bcl-2* expression remained unchanged. Consistent with this interpretation, JC-1 staining confirmed mitochondrial depolarization, supporting the conclusion that NSI-5 primarily promotes apoptosis through mitochondrial collapse and downstream intrinsic pathway activation in HL-60 cells rather than through ROS-mediated oxidative damage.

NSI-5 induced robust intrinsic apoptotic signaling in HL-60 cells, which are p53-deficient, demonstrating that apoptosis can occur in the absence of functional p53. In addition to HL-60, NSI-5 was evaluated in KG1a and K562 (mutant p53) and MV4-11 (wild-type p53) cells. Variability in sensitivity among these models suggests that response to NSI-5 is not solely dependent on TP53 status and may involve additional molecular determinants. However, because functional inhibition or direct mechanistic comparisons were not performed, our data support but do not definitively establish a p53-independent mechanism.

The ability of NSI-5 to induce apoptosis in a p53-deficient context is noteworthy, given the high frequency of TP53 alterations in acute myeloid leukemia and their association with chemoresistance [19]. These findings suggest that NSI-5 may retain activity in p53-compromised settings, although further validation in genetically defined systems is required. The potential involvement of alternative p53 family members, such as p73, remains speculative and warrants further investigation.

Several limitations should be noted. COX-independence was not directly evaluated, as no COX enzymatic assays were performed, and therefore remains inferred. In addition, mechanistic insights rely primarily on mRNA expression data without protein-level validation. Mechanistic analyses were largely confined to the HL-60 cell line, which may limit generalizability across AML subtypes. Furthermore, the study did not assess toxicity in normal hematopoietic cells or include in vivo validation. These aspects warrant further investigation.

## 4. Materials and Methods

### 4.1. Chemical Synthesis

Melting points were recorded using an RD-MP2 digital capillary melting-point apparatus (REACH Devices, Boulder, CO, USA). The NMR spectra were obtained from Bruker AVANCE-III 600 MHz and AVANCE-III HD 850 MHz spectrometers (Bruker, Rheinstetten, Germany), as well as a Spinsolve 80 MHz benchtop NMR instrument (Magritek, Aachen, Germany). The chemical shifts (δ) are reported in ppm relative to TMS as an internal reference (King Fahd Center for Medical Research and Faculty of Science, King Abdulaziz University, Jeddah, Saudi Arabia). LC/MS analyses were conducted on an Agilent 6320 Ion Trap HPLC–ESI-MS/DAD system (Agilent Technologies, Santa Clara, CA, USA) under the following conditions: The analytes were separated using a Macherey-Nagel Nucleodur-C18 column (150 mm length × 4.6 mm i.d., 5 μm) (Macherey-Nagel GMBH & Co. KG, Duren, Germany). The mobile phase was isocratic elution with an acetonitrile and 0.01 formic acid mixture in water (80:20, *v*/*v*). The flow rate was 0.4 mL/min; the run time was 20 min. Purities were reported as percentages based on the peak areas at a wavelength of 280 nm. High-resolution mass spectrometry (HRMS) was performed in the Faculty of Science, King Abdulaziz University, on an Impact II™ Q-TOF spectrometer (Bruker Daltonics GmbH, Bremen, Germany). Column chromatography was also performed using silica gel 60 (particle size 0.06–0.20 mm; Merck KGaA, Darmstadt, Germany).

### 4.2. General Methods for Synthesis of NSI- (1–9) Derivatives

With a cooled solution of the corresponding NSAID (3.63 mmol) in 15 mL of DCM, triethylamine (9.81 mmol) and EDCI HCl (5.45 mmol) were added (Scheme 1). The mixture was stirred at 0 °C for thirty minutes, and benzylamine (3.63 mmol) was added gradually and the reaction medium stirred at RT overnight. The reaction was quenched with saturated ammonium chloride and poured into a separatory funnel. The organic layer was separated, and the aqueous layer was extracted three times with ethyl acetate. The collected organic layer was washed thrice with 1 N HCl, followed by a NaHCO_3_ solution, then with water and brine. The dried organic phase was dried over anhydrous Na_2_SO_4_ and filtered, and the solvent was evaporated.

#### 4.2.1. N-Benzyl-2-(4-isobutylphenyl)propanamide (NSI-1)

NSI-1 was synthesized using ibuprofen following the general procedure. The crude product was purified by flash chromatography using a gradient hexane-ethyl acetate system to produce the pure product as white wax (39.8%), which has the following characteristic characterization profile: mp = 78.5–79 °C. 1H NMR (850 MHz, DMSO-d6) d 8.42 (t, J = 5.97 Hz, 1H), 7.22–7.28 (m, 3H), 7.18–7.22 (m, 1H), 7.11–7.16 (m, 2H), 7.09 (d, J = 8.30 Hz, 2H), 4.24 (t, J = 5.71 Hz, 2H), 3.63 (q, J = 7.27 Hz, 1H), 2.41 (d, J = 7.27 Hz, 2H), 1.81 (td, J = 6.75, 13.49 Hz, 1H), 1.34 (d, J = 7.27 Hz, 3H), 0.86 (d, J = 6.75 Hz, 5H). 13C NMR (214 MHz, DMSO-d6) d 173.9, 140.0, 140.0, 139.7, 129.2, 128.6, 127.5, 127.4, 127.1, 45.2, 44.7, 42.4, 30.1, 22.6, 19.0; RT = 10.1 min; *m*/*z* 296.1 [M+H]+.

#### 4.2.2. N-Benzyl-2-(2-((2,6-dichlorophenyl)amino)phenyl)acetamide (NSI-2)

NSI-2 was synthesized using diclofenac following the general procedure. The crude product was purified by flash chromatography, and the product was eluted using a 9:1 hexane-ethyl acetate mobile phase. The product was a white pinkish solid (0.252 g) with the following characterization profile: mp = 152.6 °C. 1H NMR (DMSO-d6, 850 MHz) δ 8.85 (br t, 1H, J = 5.7 Hz), 8.33 (s, 1H), 7.52 (d, 2H, J = 8.3 Hz), 7.3 (m, 2H), 7.2–7.3 (m, 4H), 7.17 (t, 1H, J = 8.0 Hz), 7.05 (t, 1H, J = 7.5 Hz), 6.86 (t, 1H, J = 7.3 Hz), 6.31 (d, 1H, J = 7.8 Hz), 4.32 (d, 2H, J = 6.2 Hz), 3.66 (s, 2H). 13C NMR (DMSO-d6, 214 MHz) δ 172.0, 143.3, 137.5, 131.4, 131.1, 129.7, 128.2, 126.4, 123.7, 121.1, 116.3, 61.1, 55.4, 37.6, 14.6; LCMS RT = 9.2 min; *m*/*z* 386 [M+H]+.

#### 4.2.3. (R)-N-Benzyl-2-(6-methoxynaphthalen-2-yl)propanamide (NSI-3)

NSI-3 was synthesized using naproxen following the general procedure. The crude was crystallized from methanol to yield the product as white solid (22%) with the following characterization profile: mp = 138–140 °C; 1H NMR (DMSO-d6, 850 MHz) δ 8.50 (br t, 1H, J = 6.0 Hz), 7.77 (br d, 1H, J = 8.8 Hz), 7.76 (br d, 1H, J = 8.8 Hz), 7.73 (s, 1H), 7.47 (dd, 1H, J = 1.6, 8.3 Hz), 7.28 (d, 1H, J = 2.6 Hz), 7.25 (t, 2H, J = 7.5 Hz), 7.20 (t, 1H, J = 7.3 Hz), 7.1–7.2 (m, 3H), 4.26 (d, 2H, J = 6.2 Hz), 3.87 (s, 3H), 3.80 (q, 1H, J = 7.3 Hz), 1.44 (d, 3H, J = 7.3 Hz); C NMR; RT = 6.4 min; *m*/*z* 320.1 [M+H]+.

#### 4.2.4. N-Benzyl-2-(2-fluoro-[1,1′-biphenyl]-4-yl)propanamide (NSI-4)

NSI-4 was synthesized using flurbiprofen following the general procedure. The crude product was a white solid (20%) with the following characteristics: mp = 126–127 °C; 1H NMR (DMSO-d6, 850 MHz) δ 8.56 (t, 1H, J = 6.0 Hz), 7.54 (d, 2H, J = 7.8 Hz), 7.5–7.5 (m, 3H), 7.41 (t, 1H, J = 7.2 Hz), 7.30 (t, 2H, J = 7.6 Hz), 7.27 (s, 1H), 7.26 (s, 1H), 7.2–7.2 (m, 1H), 7.19 (d, 2H, J = 7.3 Hz), 4.2–4.3 (m, 2H), 3.76 (q, 1H, J = 7.1 Hz), 1.41 (d, 3H, J = 7.3 Hz). 13C NMR (DMSO-d6, 214 MHz) δ 173.2, 159.9, 158.7, 144.6, 144.6, 139.9, 135.5, 131.0, 131.0, 129.2, 129.1, 128.7, 128.2, 127.6, 127.3, 126.9, 126.8, 124.3, 124.3, 115.4, 115.3, 45.1, 42.6, 18.8; RT = 7.1 min; *m*/*z* 344.1 [M+H]+.

#### 4.2.5. (Z)-N-Benzyl-2-(5-fluoro-2-methyl-1-(4-(methylsulfinyl)benzylidene)-1H-inden-3-yl)acetamide (NSI-5)

NSI-5 was synthesized using sulindac following the general procedure. The crude was crystallized from ACN/H_2_O to yield the product as yellow solid (26.5 mg) with the following characterization profile: mp = 164–165 °C; 1H NMR (850 MHz, DMSO-d6) d 8.64 (t, J = 5.97 Hz, 1H), 7.77–7.82 (m, 2H), 7.69–7.75 (m, J = 8.30 Hz, 2H), 7.36 (s, 1H), 7.28–7.33 (m, 2H), 7.21–7.28 (m, 2H), 7.17 (dd, J = 5.19, 8.30 Hz, 1H), 7.13 (dd, J = 2.34, 9.08 Hz, 1H), 6.72 (dt, J = 2.60, 8.82 Hz, 1H), 4.29 (d, J = 6.23 Hz, 2H), 3.52 (s, 2H), 2.83 (s, 3H), 2.20 (s, 3H). 13C NMR (214 MHz, DMSO-d6) d 169.3, 163.6, 162.4, 147.8, 147.7, 146.7, 141.0, 139.9, 139.1, 138.3, 134.1, 130.4, 130.0, 130.0, 129.8, 128.7, 127.7, 127.3, 124.4, 123.5, 110.9, 110.8, 106.8, 106.7, 43.6, 42.8, 33.2, 21.2, 14.6, 10.9; RT = 4.4 min; *m*/*z* 446 [M+H]+.

#### 4.2.6. 2-(3-benzoylphenyl)-N-Benzylpropanamide (NSI-6)

NSI-6 was synthesized using ketoprofen following the general procedure. The crude was purified by flash chromatography (2:1 hexane-ethyl acetate) to yield the product as white solid (27.5%) with the following characterization profile: mp = 100.5–101 °C; 1H NMR (DMSO-d6, 850 MHz) δ 8.49 (t, 1H, J = 6.0 Hz), 7.68 (s, 1H), 7.66 (dd, 2H, J = 1.6, 8.3 Hz), 7.61 (t, 1H, J = 7.2 Hz), 7.58 (d, 1H, J = 7.3 Hz), 7.53 (d, 1H, J = 7.4 Hz), 7.49 (t, 2H, J = 7.5 Hz), 7.45 (t, 1H, J = 8.0 Hz), 7.19 (t, 2H, J = 7.7 Hz), 7.1 (m, 1H), 7.08 (d, 2H, J = 6.7 Hz), 4.1–4.3 (m, 2H), 3.72 (q, 1H, J = 6.7 Hz), 1.33 (d, 3H, J = 7.3 Hz). 13C NMR (DMSO-d6, 214 MHz) δ 196.2, 173.4, 143.2, 139.9, 137.5, 137.4, 133.2, 132.1, 130.1, 129.1, 129.0, 129.0, 128.7, 128.6, 127.5, 127.2, 45.4, 42.5, 18.9; RT = 6.2 min; *m*/*z* 344.1 [M+H]+.

#### 4.2.7. N-Benzyl-2-(1,8-diethyl-1,3,4,9-tetrahydropyrano[3,4-b]indol-1-yl)acetamide (NSI-7)

NSI-7 was synthesized using etodolac following the general procedure. The crude was purified by flash chromatography (2:1 hexane-ethyl acetate) to yield the product as white solid (46.5%) with the following characterization profile: mp = 121.4–122 °C; 1H NMR (DMSO-d6, 850 MHz) δ 10.52 (s, 1H), 8.14 (t, 1H, J = 6.0 Hz), 7.23 (t, 3H, J = 7.3 Hz), 7.2 (m, 1H), 7.17 (d, 2H, J = 7.3 Hz), 6.92 (t, 1H, J = 7.1 Hz), 6.9–6.9 (m, 1H), 4.33 (dd, 1H, J = 6.2, 15.1 Hz), 4.25 (dd, 1H, J = 5.7, 15.1 Hz), 3.9–4.0 (m, 2H), 2.91 (d, 1H, J = 14.0 Hz), 2.83 (q, 2H, J = 7.4 Hz), 2.75 (d, 1H, J = 14.0 Hz), 2.6–2.7 (m, 1H), 2.6 (m, 1H), 2.04 (q, 2H, J = 7.3 Hz), 1.25 (t, 3H, J = 7.5 Hz), 0.67 (t, 3H, J = 7.5 Hz). 13C NMR (DMSO-d6, 214 MHz) δ 169.8, 139.9, 137.2, 134.9, 128.6, 127.4, 127.0, 127.0, 126.6, 120.1, 119.2, 115.9, 107.4, 76.0, 60.4, 44.4, 42.5, 31.0, 24.2, 22.4, 14.9, 8.3; RT = 8.3 min; *m*/*z* 377.1 [M+H]+.

#### 4.2.8. N-Benzyl-2-(1-(4-chlorobenzoyl)-5-methoxy-2-methyl-1H-indol-3-yl)acetamide (NSI-8)

NSI-8 was synthesized using indomethacin following the general procedure. The crude was purified by flash chromatography (60:40 hexane-ethyl acetate) to yield the product as a pale yellow solid (32%) with the following characterization profile: mp = 165.4–166 °C; 1H NMR (850 MHz, DMSO-d6) d 8.53 (s, 1H), 7.70 (d, J = 1.00 Hz, 2H), 7.65 (d, J = 1.00 Hz, 2H), 7.26–7.36 (m, 2H), 7.18–7.26 (m, 2H), 7.14 (d, J = 2.59 Hz, 1H), 6.97 (d, J = 8.82 Hz, 1H), 6.72 (dd, J = 2.59, 8.82 Hz, 1H), 4.29 (d, J = 5.71 Hz, 2H), 3.74 (s, 3H), 3.59 (s, 2H), 2.23 (s, 2H). 13C NMR (214 MHz, DMSO-d6) d 169.9, 168.3, 156.0, 140.0, 138.0, 135.7, 134.8, 131.6, 131.3, 130.8, 129.5, 128.7, 127.7, 127.2, 115.1, 114.8, 111.9, 102.2, 55.9, 42.7, 31.6, 14.6, 13.9; RT = 7.6 min; *m*/*z* 447 [M+H]+.

#### 4.2.9. 5-Benzoyl-N-Benzyl-2,3-dihydro-1H-pyrrolizine-1-carboxamide (NSI-9)

NSI-9 was synthesized using ketorolac following the general procedure. The crude was crystallized from acetone to yield the product as white solid (35.7 mg) with the following characterization profile: mp = 180 °C; 1H NMR (850 MHz, DMSO-d6) d 8.80 (t, J = 5.97 Hz, 1H), 7.72–7.81 (m, 2H), 7.56–7.67 (m, 1H), 7.49–7.56 (m, 2H), 7.34 (t, J = 7.53 Hz, 2H), 7.28 (d, J = 7.78 Hz, 1H), 7.23–7.27 (m, 1H), 6.77 (d, J = 4.15 Hz, 1H), 6.04 (d, J = 4.15 Hz, 1H), 4.42 (ddd, J = 5.71, 8.56, 11.68 Hz, 1H), 4.35 (d, J = 5.71 Hz, 1H), 4.28–4.34 (m, 2H), 4.03 (dd, J = 5.71, 8.82 Hz, 1H), 2.75–2.88 (m, 1H), 2.63–2.75 (m, 1H). 13C NMR (214 MHz, DMSO-d6) d 183.9, 170.9, 145.8, 139.7, 139.4, 131.9, 128.9, 128.8, 128.8, 127.7, 127.3, 126.5, 125.0, 102.8, 48.3, 43.3, 42.9, 31.3; LCMS RT = 5.9 min; *m*/*z* 345.1 [M+H]+. The characterization of all NSI compounds is shown in Supplementary Materials.

### 4.3. Methods for Biological Experiments on Leukemic Cell Lines

#### 4.3.1. Cell Culture

The leukemic cell lines (HL-60, MV4-11, KG1a, and K562) were purchased from commercial cell line banks. All leukemic cell lines were cultured in Roswell Park Memorial Institute (RPMI) medium (Gibco, Thermo Fisher Scientific, Waltham, MA, USA) supplemented with 10% fetal bovine serum (FBS) (Gibco, Thermo Fisher Scientific, Waltham, MA, USA) and 0.1% penicillin/streptomycin antibiotic (Gibco, Thermo Fisher Scientific, Waltham, MA, USA). Cells were incubated in 5% CO_2_ at 37 °C.

#### 4.3.2. CellTiter-Blue Cell Viability Assay

Cell viability of leukemic cell lines following treatment with NSI-(1-9) compounds was measured using the Cell-Titer^®^-Blue cell viability assay kit (Promega, Madison, WI, USA), as previously described [20,21]. Briefly, 1 × 104 cells were seeded in a 96-well plate and treated with different concentrations of NSI compounds ranging from 0.01 to 40 μM in triplicate. The cells were then incubated for 48 h at 37 °C in 5% CO_2_. After treatment, 20 μL of CellTiter^®^-Blue reagent was added to each well, and the plates were further incubated for 2 h to allow fluorescence development. Fluorescence intensity was measured using a SpectraMax^®^ i3 Multi-Mode microplate reader at an excitation/emission wavelength of 540/590 nm (Molecular Devices, San Diego, CA, USA). The data were plotted against drug concentrations to calculate the half-maximal inhibitory concentration (IC50 value) using a nonlinear regression model in GraphPad Prism 10.1.1 software (GraphPad Software Inc., Boston, MA, USA).

#### 4.3.3. Detection of Apoptosis Cell Death

Apoptosis was detected using an annexin V-FITC apoptosis detection kit (BD Pharmingen, San Diego, CA, USA) [21,22]. Briefly, 1.5 × 10^5^ of leukemic cell lines were seeded in a 6-well plate with appropriate NSI-5 concentrations (1, 2, and 3 μM) in 5% CO_2_ at 37 °C for 48 h. Next, cells were washed twice with cold PBS (1×; Gibco, Thermo Fisher Scientific, Waltham, MA, USA) and then resuspended in 1× Binding Buffer. Finally, the cells were analyzed by acquiring a minimum of 10,000 events using a BD FACSAria III flow cytometer (BD Biosciences, San Diego, CA, USA).

#### 4.3.4. Cell Cycle Analysis

The aim of this experiment is to evaluate whether the treatment with NSI-5 compound altered the distribution of cells in the cell cycle phases [23]. Leukemic cells were seeded at a density of 3 × 105 cells/well. The cells were treated with 1 μM NSI-5 compound for 48 h in 5% CO_2_ at 37 °C. After that, the leukemic cells were collected and washed twice with PBS (1×). The cells were fixed using 70% ice cold ethanol and stored at −20 °C overnight. The following day, the fixed cells were washed twice with PBS. Thereafter, the cells were stained with propidium iodide (PI; Thermo Fisher Scientific, Waltham, MA, USA) following the manufacturer’s instructions. A minimum of 10,000 events were analyzed using a BD FACSAria III flow cytometer (BD Biosciences, San Diego, CA, USA).

#### 4.3.5. Mitochondrial Membrane Potential (MMP) Analysis

To assess the changes in MMP during apoptosis stimulated by the JC-1 dye (J-aggregate-forming cationic dye;Invitrogen™, Thermo Fisher Scientific, Waltham, MA, USA), flow cytometry was utilized [24]. After 48 h of incubating HL-60 cells with 1 μM NSI-5, the cells were collected, washed with 1× PBS, and stained with 10 µg/mL JC-1 dye. The stained cells were incubated for 10 min at 37 °C in 5% CO_2_. Immediately, the red fluorescent intensity of JC-1 aggregates (polarized mitochondrial membrane), and the green fluorescence intensity of JC-1 monomer (depolarized mitochondrial membrane), were detected at emissions of 590 nm and 535 nm, respectively, by a BD FACSAria^TM^ III flow cytometer. Then, the percentages of these variables were analyzed via BD FACSDiva 9.0.1 flow cytometry software. A total of 10,000 events were acquired for the analysis.

#### 4.3.6. Intracellular ROS Measurement

In 6-well plate, a total of 1.5 × 10^5^ of HL-60 cells were seeded and treated with 1 μM NSI-5 for 48 h. At end of this point, cells were collected and stained with the intracellular fluorogenic dye (2′,7′-dichlorofluorescin diacetate; DCFDA) (Abcam, Cambridge, UK) to measure the level of ROS generation after the addition of the test compound [25]. This experiment was performed according to the manufacturer’s instructions, and the samples were immediately analyzed using BD FACSAria^TM^ III flow cytometry (BD Biosciences, San Jose, CA, USA). At least 1 × 10^4^ events were acquired for the analysis.

#### 4.3.7. Gene Expression (RT-PCR) Analysis

Briefly, HL-60 cells were seeded at 3 × 10^6^ cells in a 75 cm^2^ flask and treated with 1 and 2 μM NSI-5 for 48 h. After collecting the cells, total RNA was extracted using a Qiagen RNeasy kit (Qiagen, Hilden, Germany) following the manufacturer’s instructions. RNA yield was quantified using a NanoDrop DeNovix DS-11 (DeNovix Inc., Wilmington, DE, USA). Next, cDNA was synthesized from RNA using a High-Capacity cDNA reverse transcription kit (Applied Biosystems™, Thermo Fisher Scientific, Waltham, MA, USA). cDNA was used for RT-PCR analysis using a Fast SYBRTM Green Master Mix (Applied Biosystems™, Thermo Fisher Scientific, Waltham, MA, USA). The mRNA expression levels of apoptosis-related genes (*Bcl-2*, *Bax*, *Bak1*, *PARP-1*, *MDM1*, and *Caspases 3*) and antioxidant-related genes (*SOD-1*, *CAT*, *GPX-1*, and *GSS*) were determined using a StepOnePlusTM real-time PCR Applied Biosystems™, Thermo Fisher Scientific, Waltham, MA, USA). The mRNA levels of the examined genes were measured relative to *GAPDH* mRNA levels. The 2^−ΔΔct^ method was employed to calculate the fold change. The primer sequences of the genes are listed in Table 2.

### 4.4. Data Analysis

Values are shown as the mean ± standard error of the mean (SEM) from at least three independent experiments (n = 3) conducted using leukemia cell lines. The IC50 values for NSI compounds against leukemia cells were estimated using dose–response curves generated by nonlinear regression analysis with the aid of GraphPad Prism version 10.1.1 (GraphPad Software Inc., Boston, MA, USA). Moreover, an unpaired Student’s *t*-test in GraphPad Prism was performed to compare NSI-5-treated cells with control cells. Differences among more than two groups were identified using one-way analysis of variance (ANOVA) and Dunnett’s post hoc test, and two-way ANOVA and Tukey’s multiple comparisons test were used to compare the mean difference in two variables between three or more groups. The value of *p* ≤ 0.05 was considered significant in all analyses.

## 5. Conclusions

This multi-parametric study demonstrates that NSI-5 exhibits pro-apoptotic activity in myeloid leukemia models, with mechanistic characterization primarily conducted in the HL-60 cell line. Combined flow cytometric and molecular analyses indicate that NSI-5 induces apoptosis associated with modulation of key regulators, including an increased *Bax*/*Bcl-2* ratio and activation of caspase-3. These effects were accompanied by a loss of mitochondrial membrane potential (ΔΨm), suggesting involvement of the intrinsic apoptotic pathway.

NSI-5 treatment was also associated with reduced intracellular reactive oxygen species (ROS) levels and downregulation of antioxidant-related transcripts, including *CAT* and *GSS*, indicating disruption of redox homeostasis. The observed molecular changes are consistent with the activation of apoptotic signaling that may occur independently of p53; however, this was not directly validated and requires further investigation.

Collectively, these findings identify NSI-5 as a promising in vitro anti-leukemic lead compound. Further studies are required to confirm these mechanisms at the protein level, evaluate activity across additional leukemia models, and assess efficacy and safety in vivo. In particular, investigation in genetically defined systems and evaluation in normal hematopoietic cells will be essential to determine therapeutic selectivity and translational potential.

## Figures and Tables

**Table 1:** Cytotoxic activity of the synthesized compounds against leukemia cell lines, expressed as IC_50_ ± SD (µM).

*Compound*	*MV4-11*	*HL-60*	*KG1a*	*K562*
***NSI-1***	32.4 ± 0.66	18.9 ± 1.10	>50	36.9 ± 0.15
***NSI-2***	>50	>50	>50	>50
***NSI-3***	2.8 ± 2.2	1.5 ± 0.74	2.4 ± 0.35	1 ± 1.42
***NSI-4***	3.5 ± 0.56	4.3 ± 0.31	5.6 ± 0.66	2.7 ± 0.21
***NSI-5***	**1.3 ± 0.67**	**0.6 ± 0.40**	**1.5 ± 0.73**	**1.3 ± 0.13**
***NSI-6***	11.8 ± 3.3	10.2 ± 1.39	9.1 ± 1.33	6.5 ± 0.72
***NSI-7***	15.7 ± 2.80	25.4 ± 3.01	18.9 ± 1.43	32.6 ± 0.46
***NSI-8***	12.2 ± 1.21	20.5 ± 0.84	>50	17.8 ± 0.63
***NSI-9***	3.2 ± 0.38	3.9 ± 0.77	3.8 ± 0.08	3.1 ± 0.10

Bold values indicate the lowest IC_50_ values (highest potency) across leukemia cell lines.

**Table 2:** List of genes and primer sequences used for RT-qPCR.

Gene Name	NCBI Gene ID	Primers
*GADPH*	2597	F: CTGGGCTACACTGAGCACC R: AAGTGGTCGTTGAGGGCAATG
*Bcl-2*	596	F: GGTGGGGTCATGTGTGTGG R: CGGTTCAGGTACTCAGTCATCC
*Bax*	581	F: CCCGAGAGGTCTTTTTCCGAG R: CCAGCCCATGATGGTTCTGAT
*Bak1*	578	F: GTTTTCCGCAGCTACGTTTTT R: GCAGAGGTAAGGTGACCATCTC
*PARP-1*	142	F: TGGAAAAGTCCCACACTGGTA R: AAGCTCAGAGAACCCATCCAC
*MDM1*	56890	F: TCAAGGCAGATGTAGAACAAGA R: ACCAAGATGAGAAGGGATGAGA
*Caspases 3*	836	F: AGAGGGGATCGTTGTAGAAGTC R: ACAGTCCAGTTCTGTACCACG
*SOD-1*	6647	F: GGTGGGCCAAAGGATGAAGAG R: CCACAAGCCAAACGACTTCC
*CAT*	847	F: TGTTGCTGGAGAATCGGGTTC R: TCCCAGTTACCATCTTCTGTGTA
*GPX-1*	2876	F: CAGTCGGTGTATGCCTTCTCG R: GAGGGACGCCACATTCTCG
*GSS*	2937	F: GGAACATCCATGTGATCCGAC R: GCCATCCCGGAAGTAAACCA

## Data Availability

The original contributions presented in this study are included in the article/Supplementary Materials. Further inquiries can be directed to the corresponding author.

## Supplementary Materials

The following supporting information can be downloaded at: https://www.mdpi.com/article/10.3390/ijms27093850/s1.
