# Supplementary figures and images for: Novel NSAID Analogs Exhibit Anti-Leukemic Activity Through Modulation of Apoptotic and Survival Pathways
Source: Int J Mol Sci. 2026 Apr 26;27(9):3850. doi: 10.3390/ijms27093850 (PMC13163830; doi:10.3390/ijms27093850)

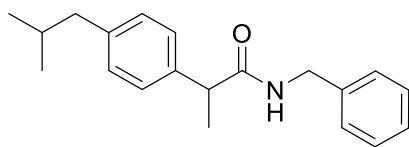

**NSI-1:**

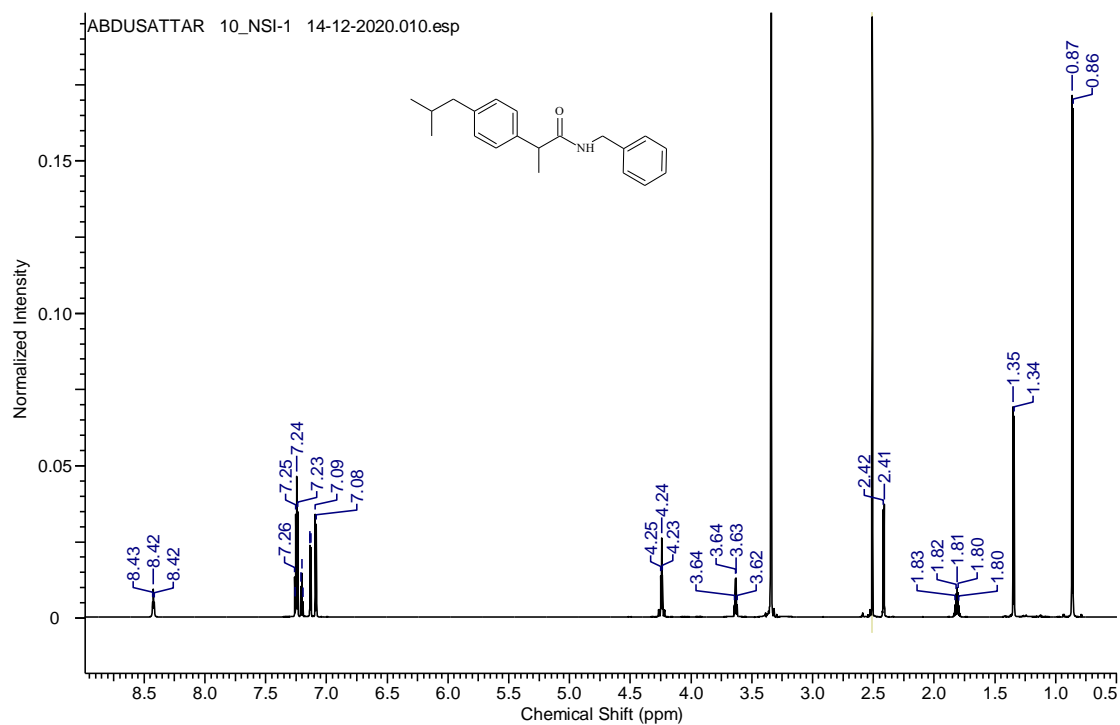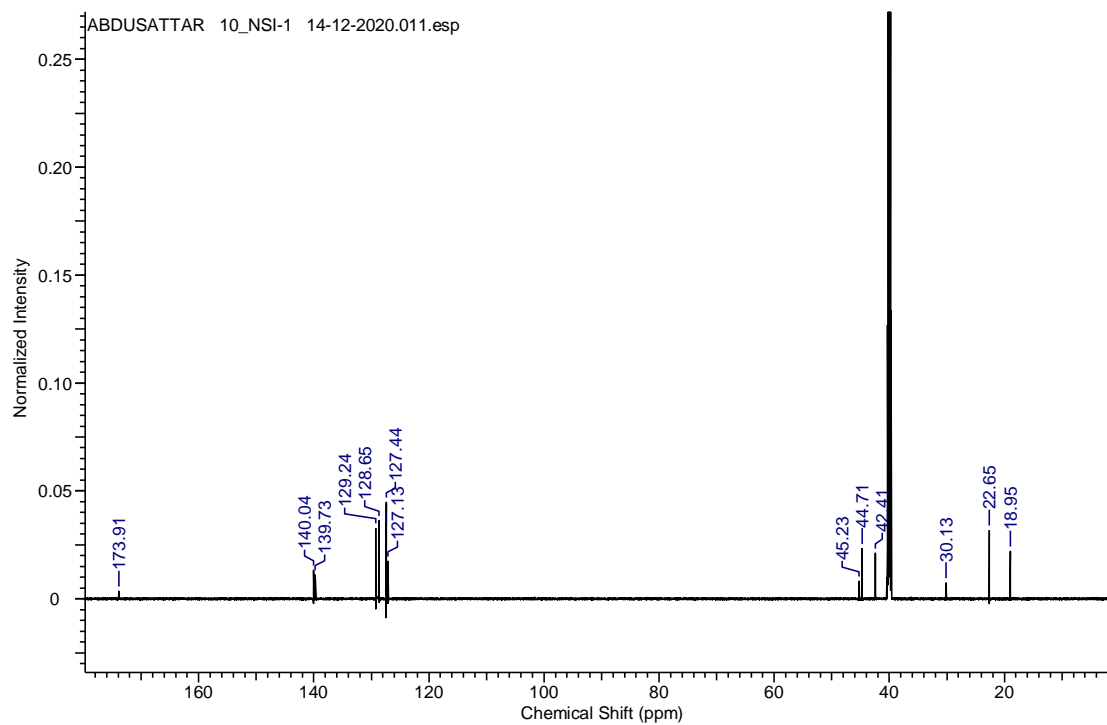

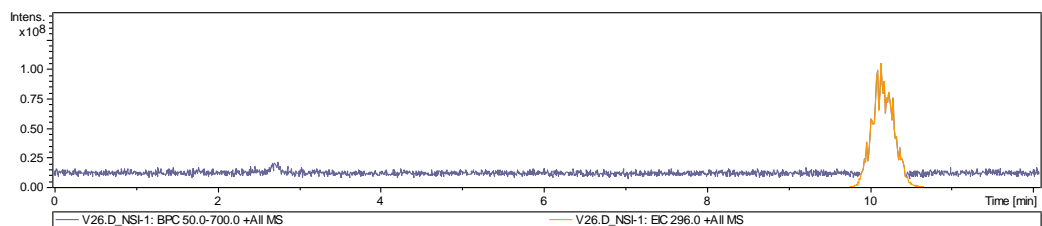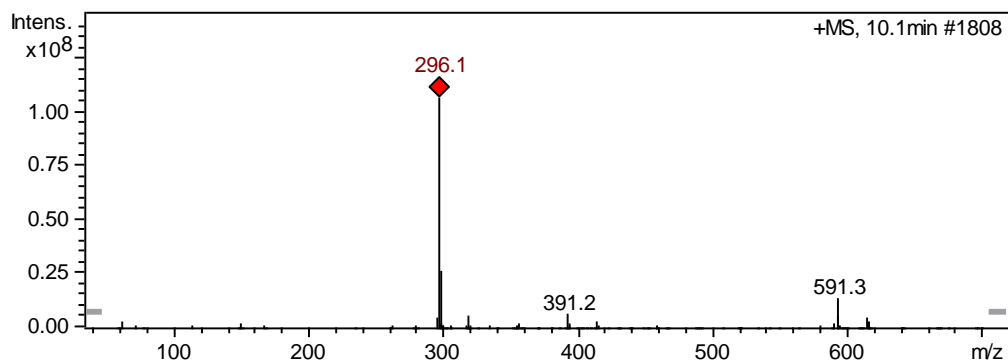

## NSI-2:

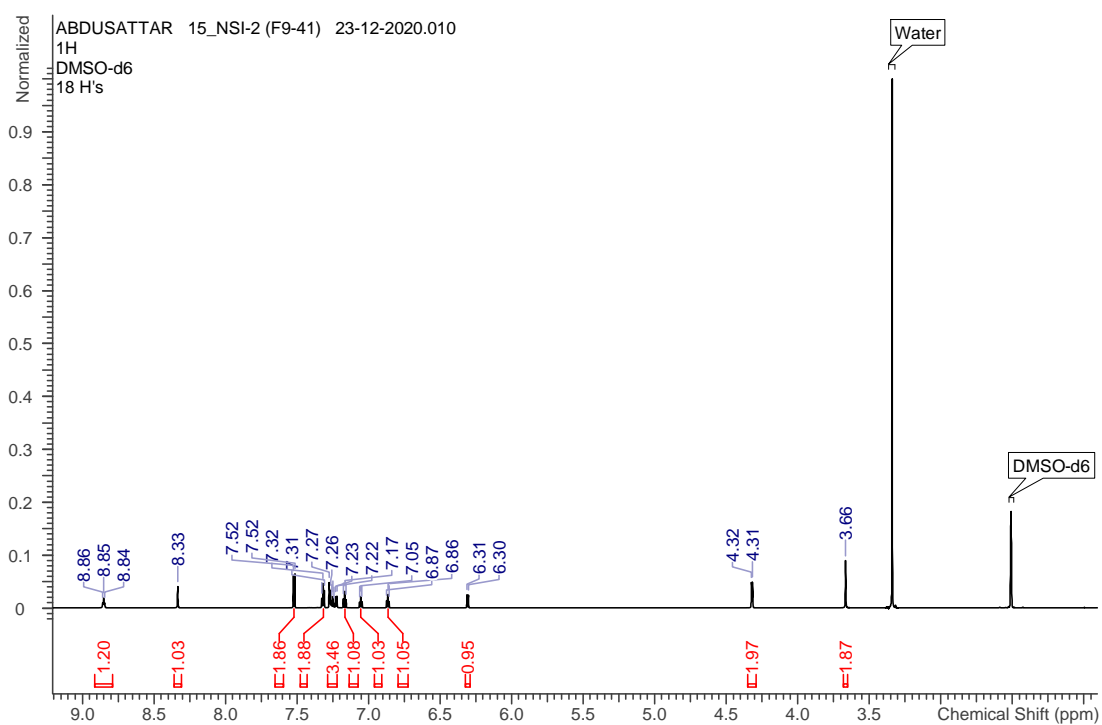

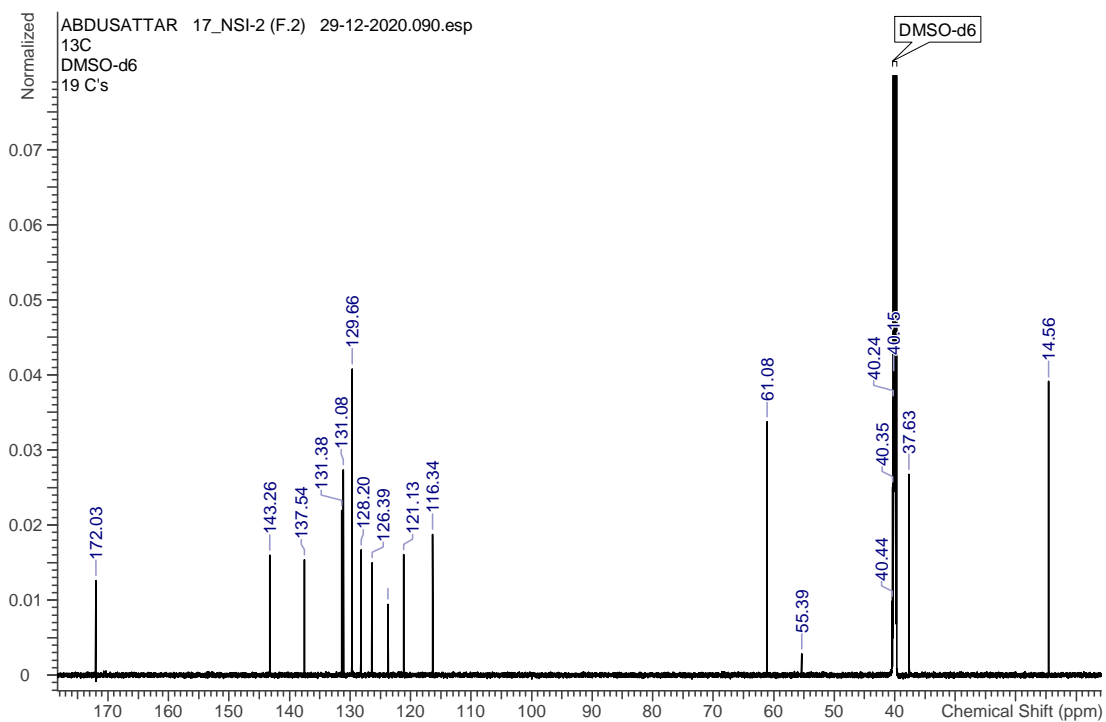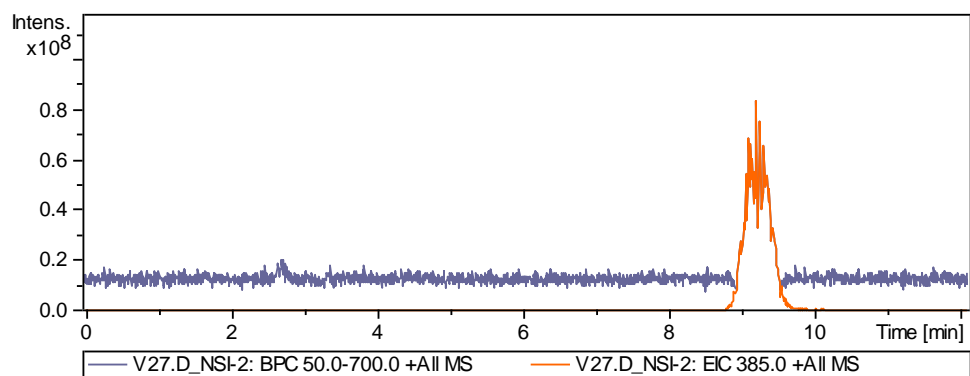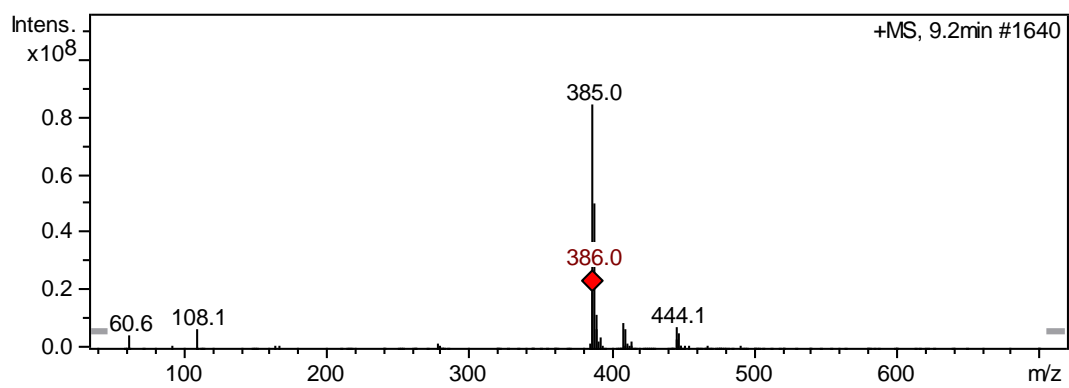

### NSI-3:

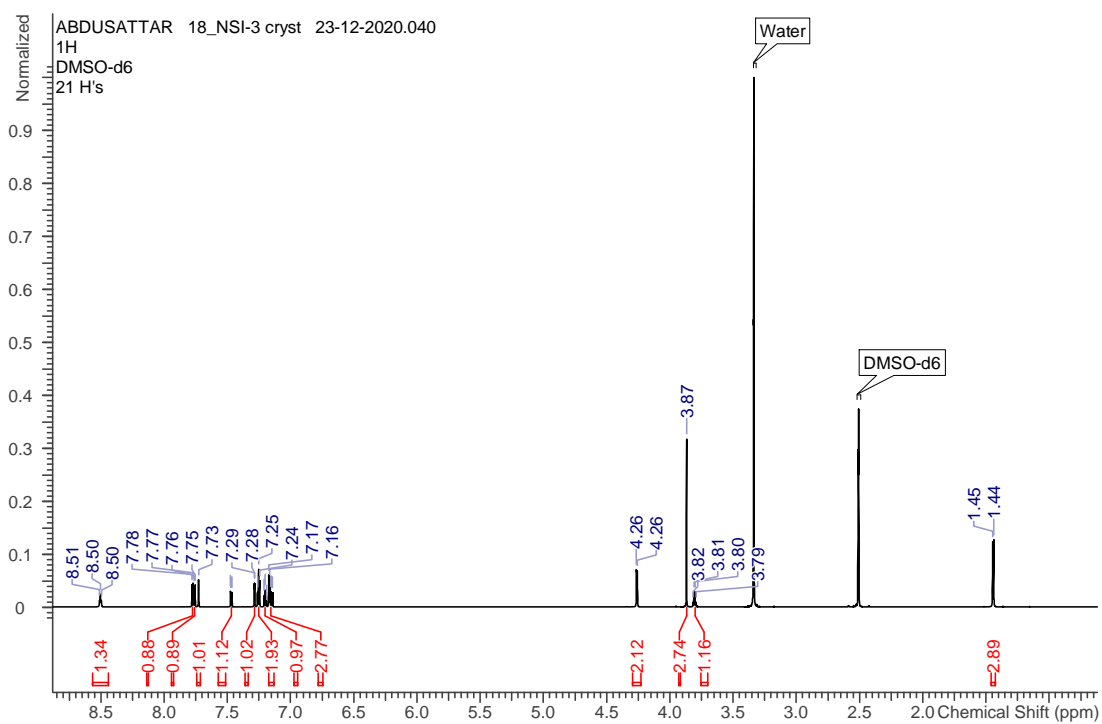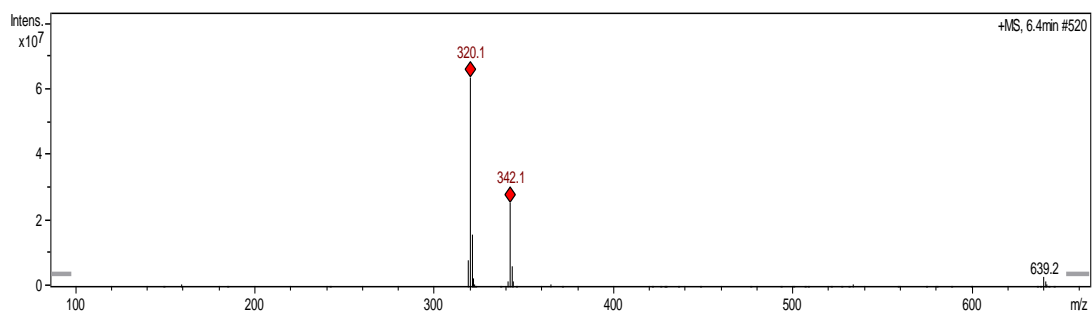

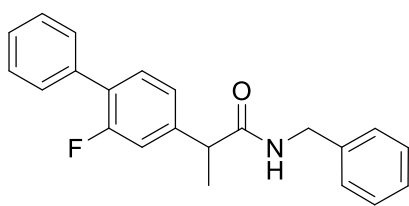

# NSI-4:

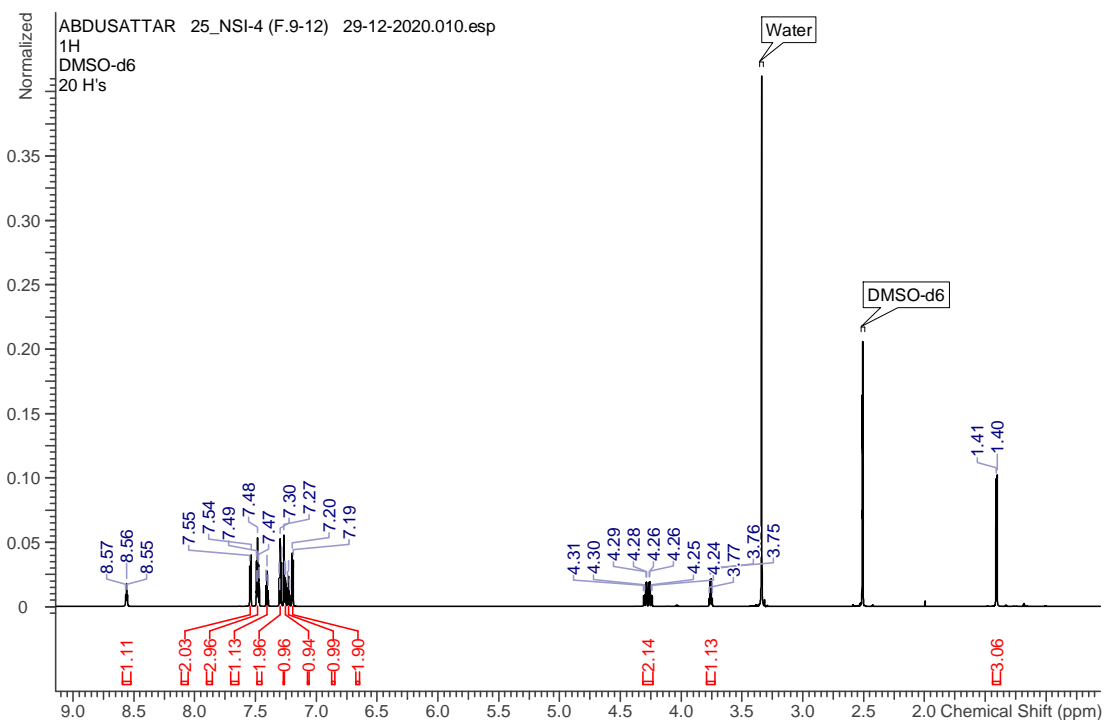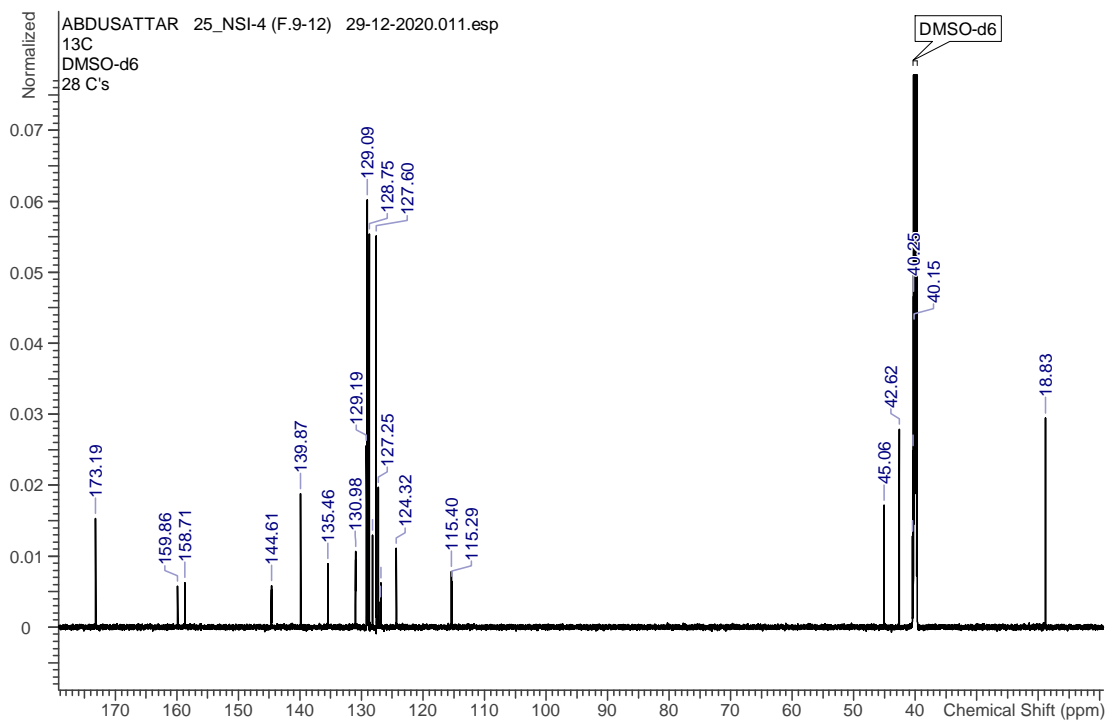

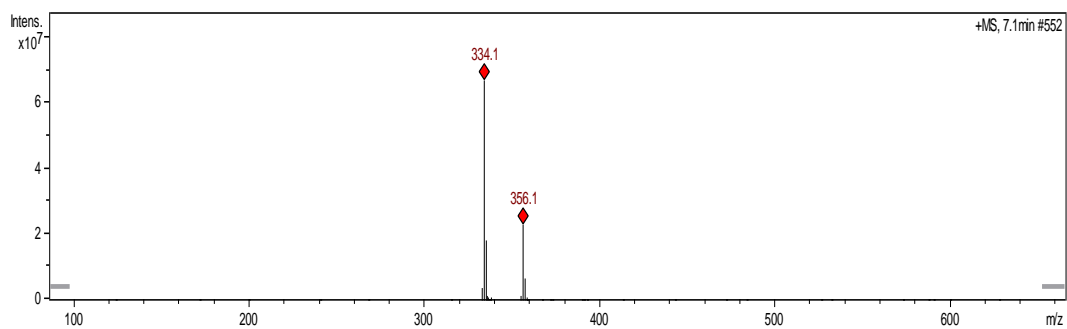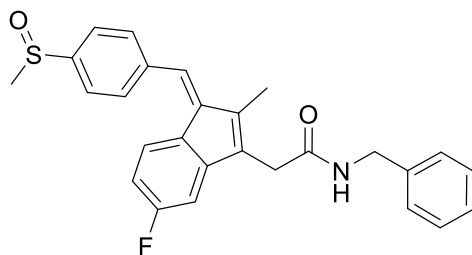

**NSI-5:**

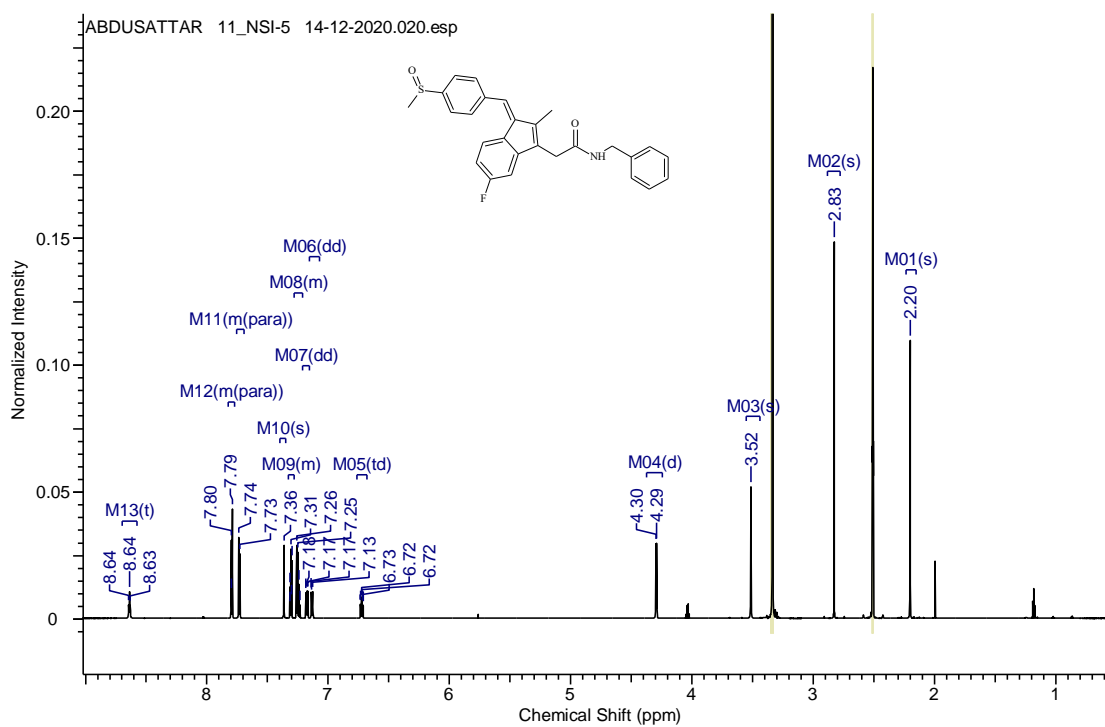

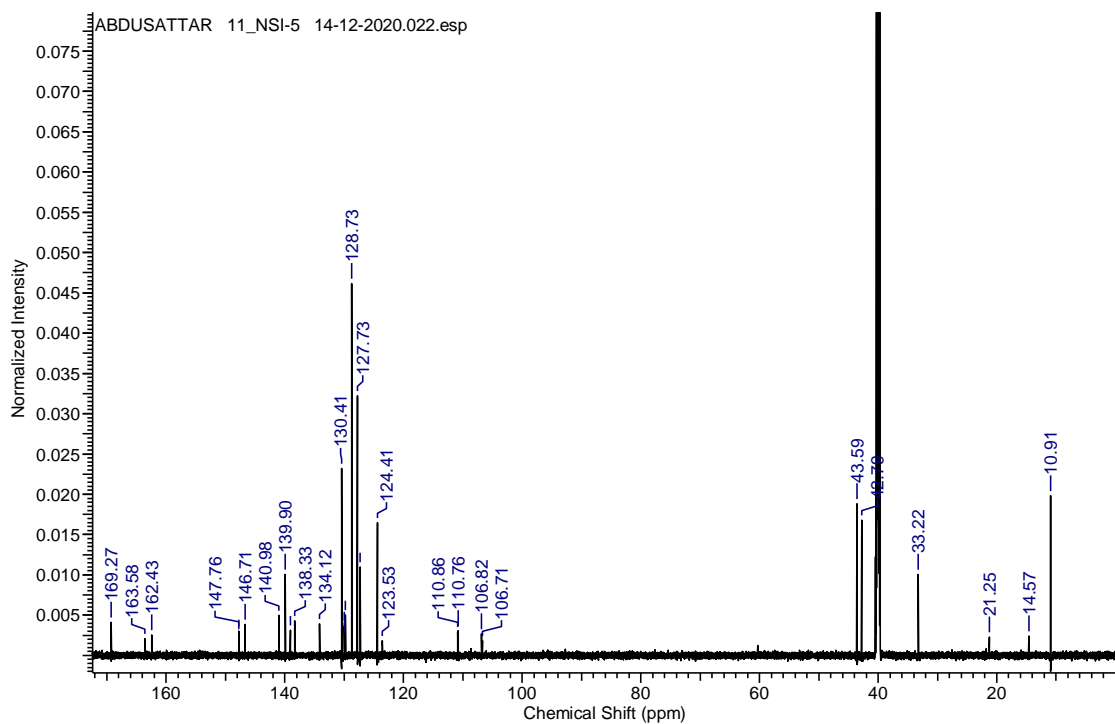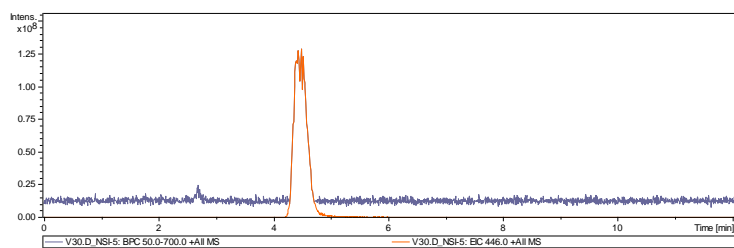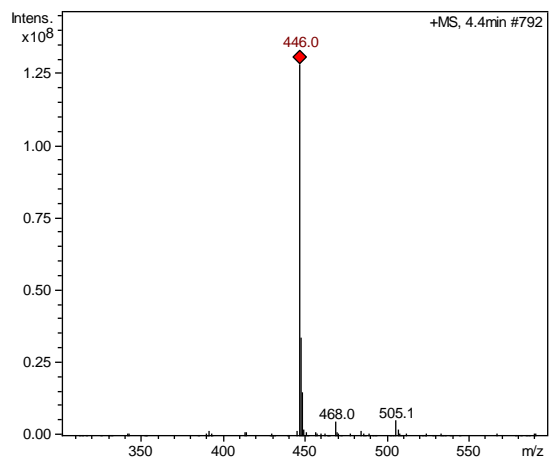

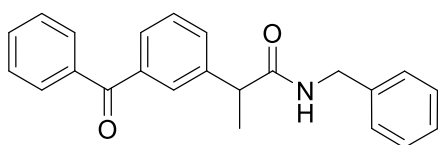

# NSI-6:

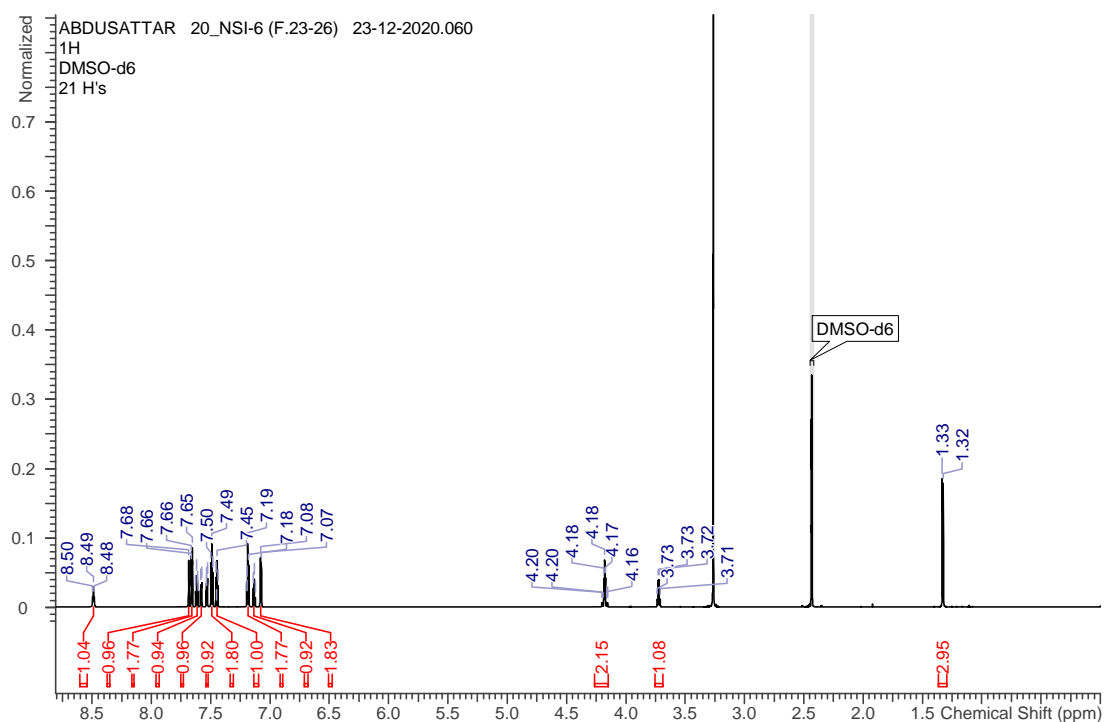

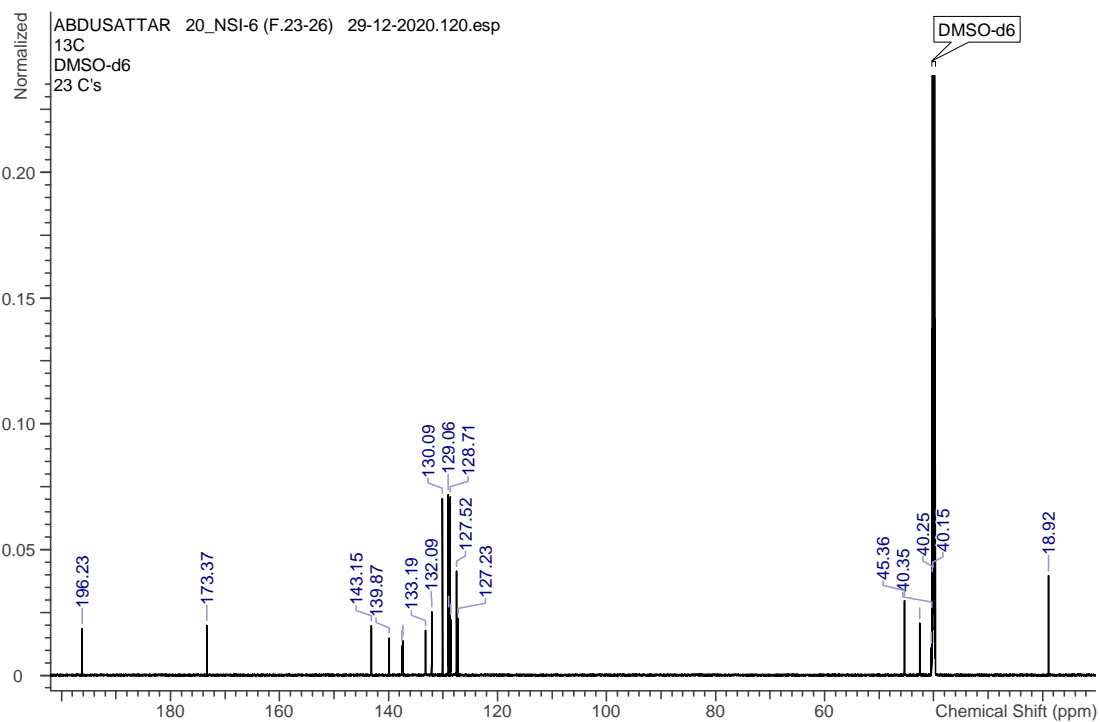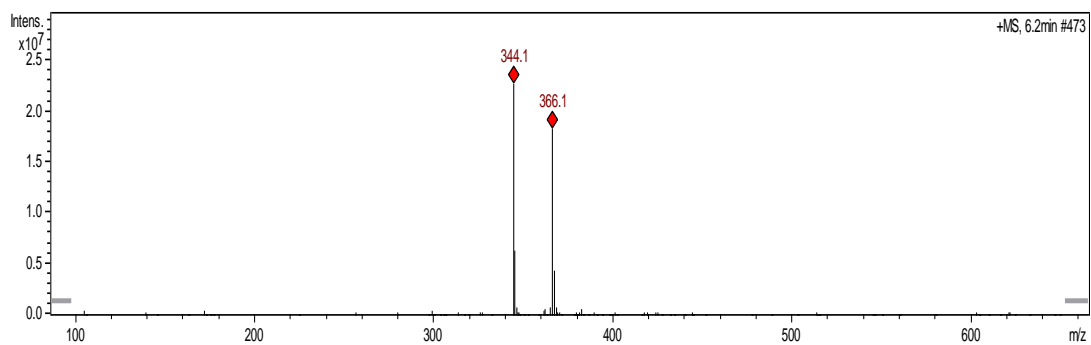

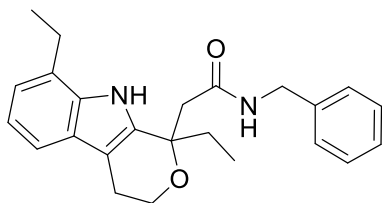

**NSI-7:**

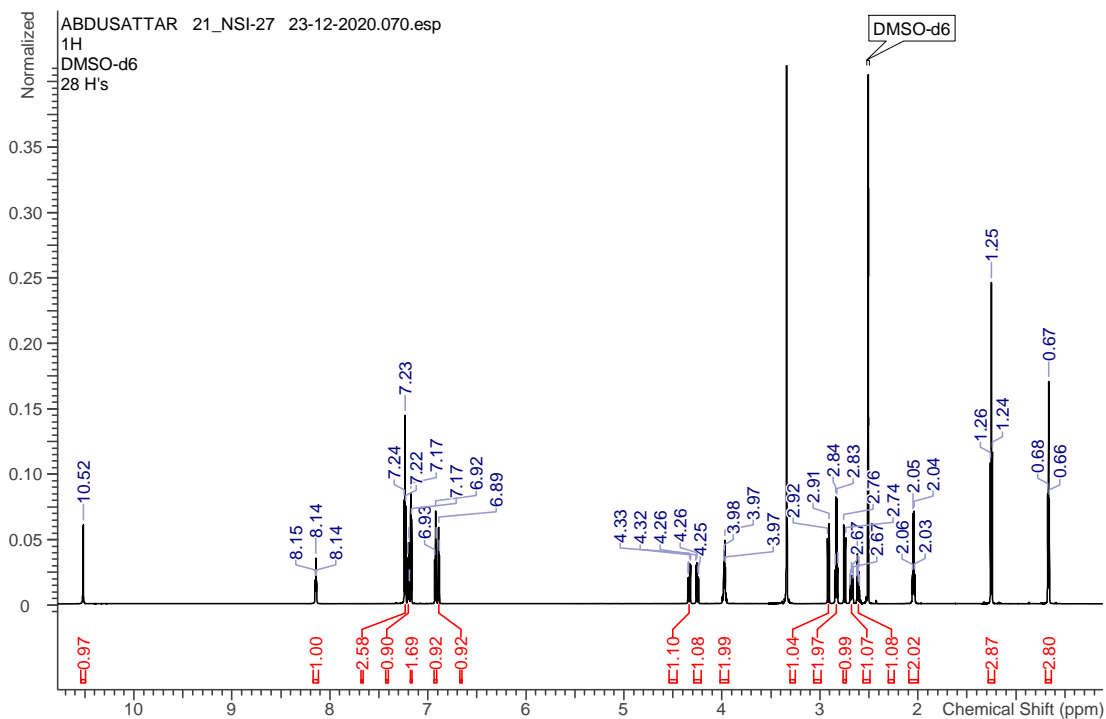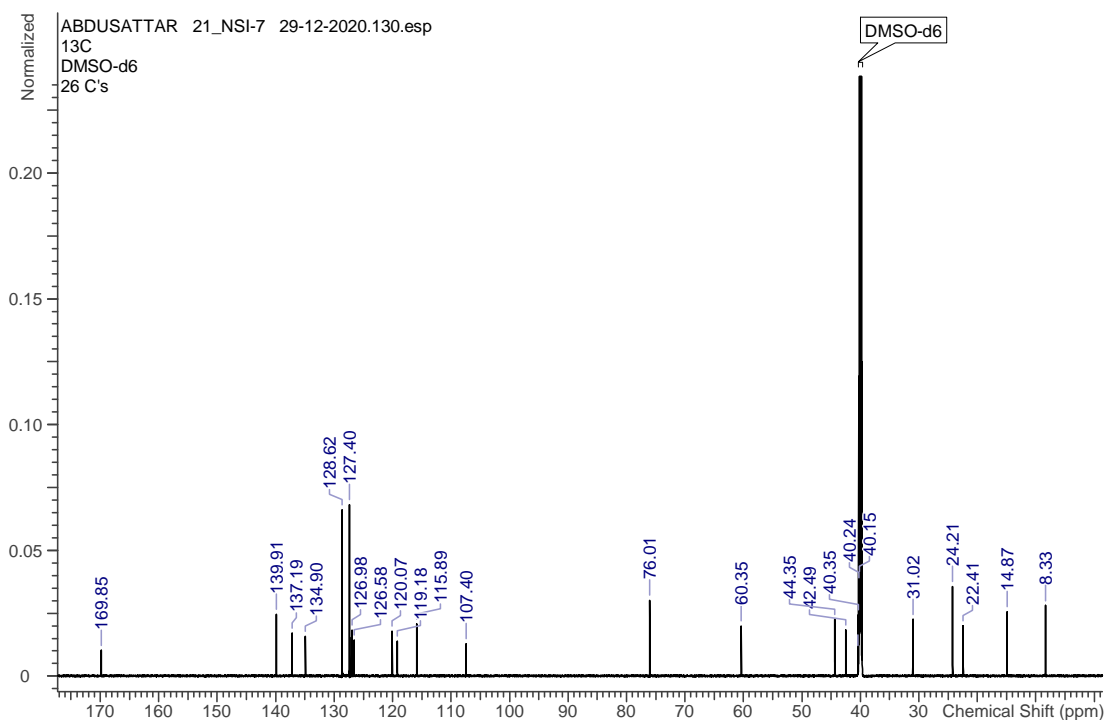

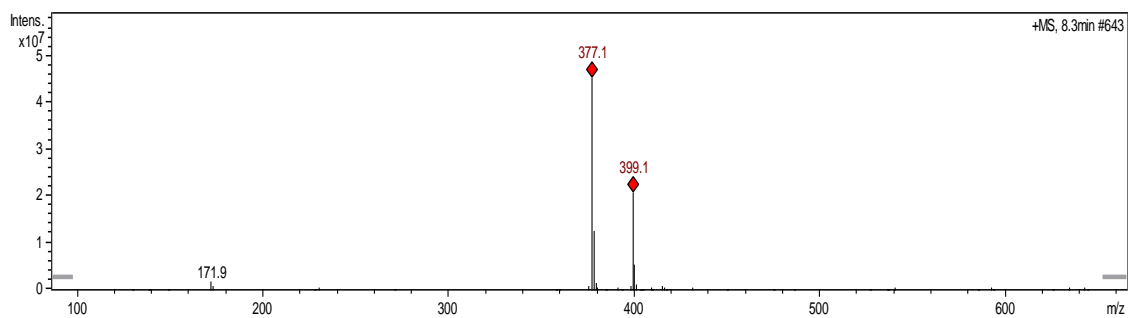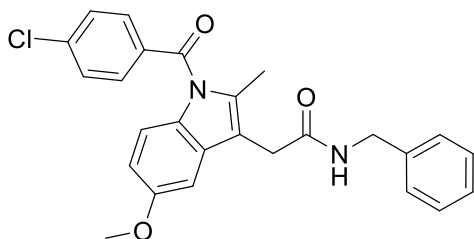

**NSI-8:**

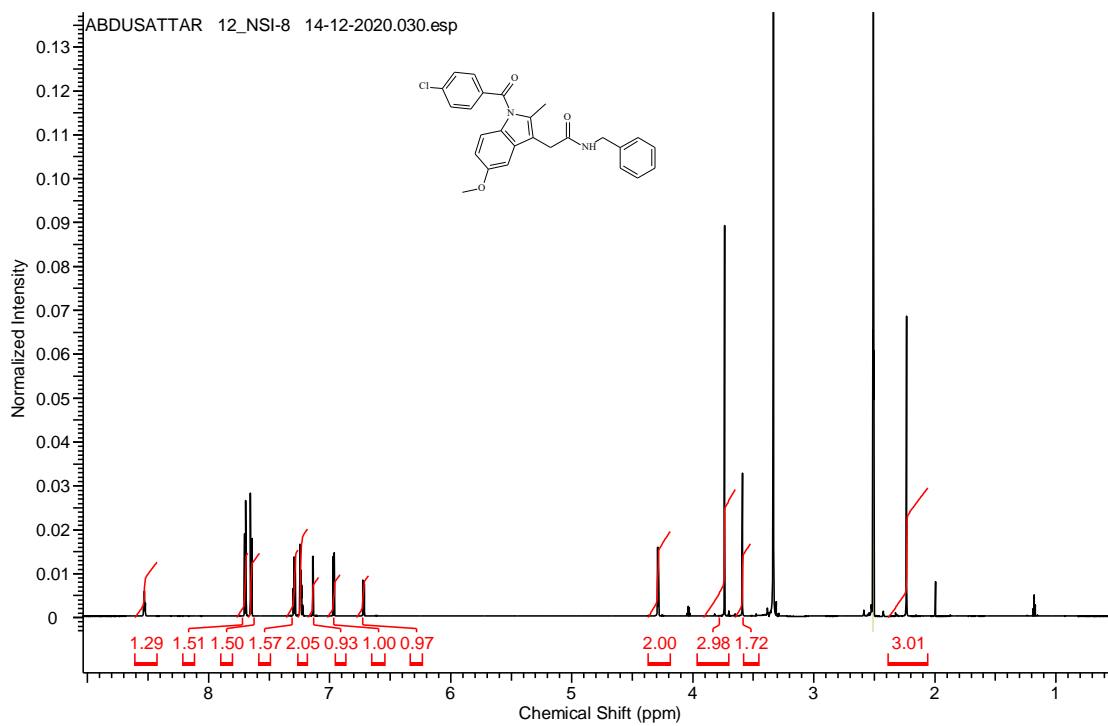

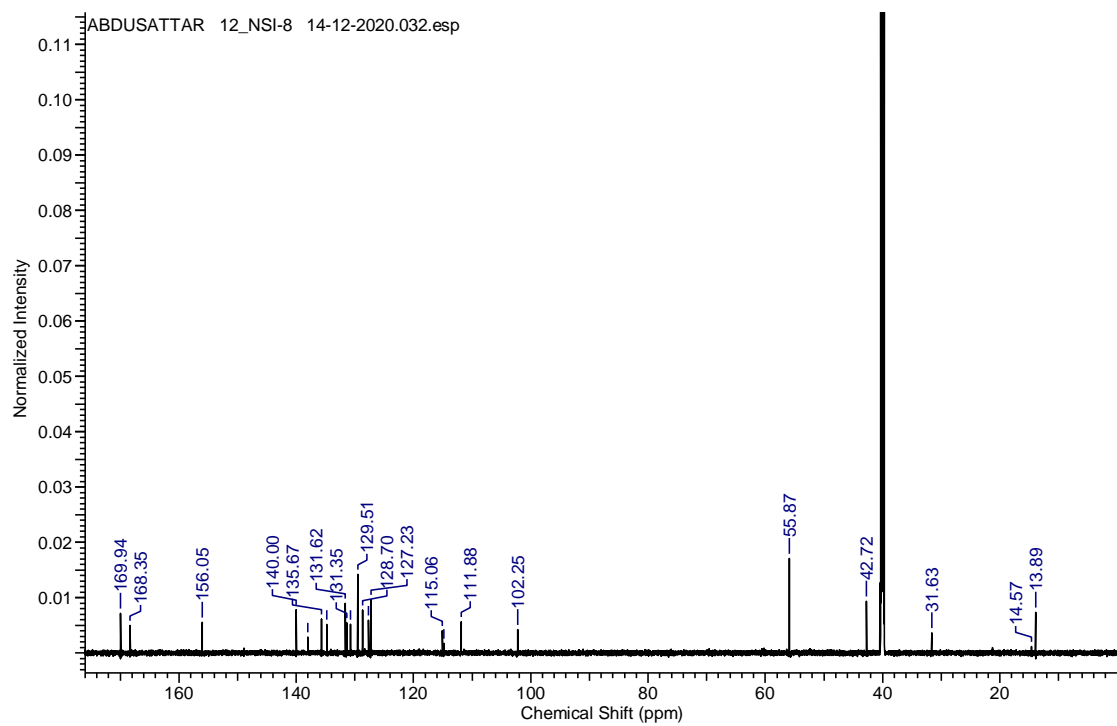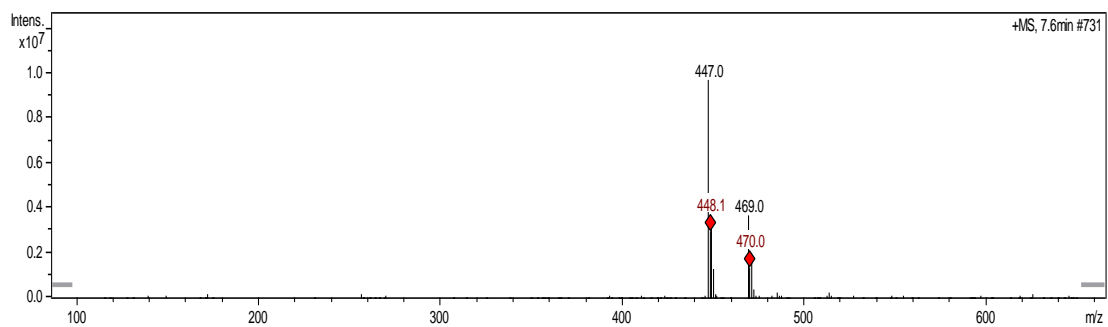

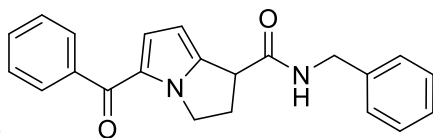

NSI-9:

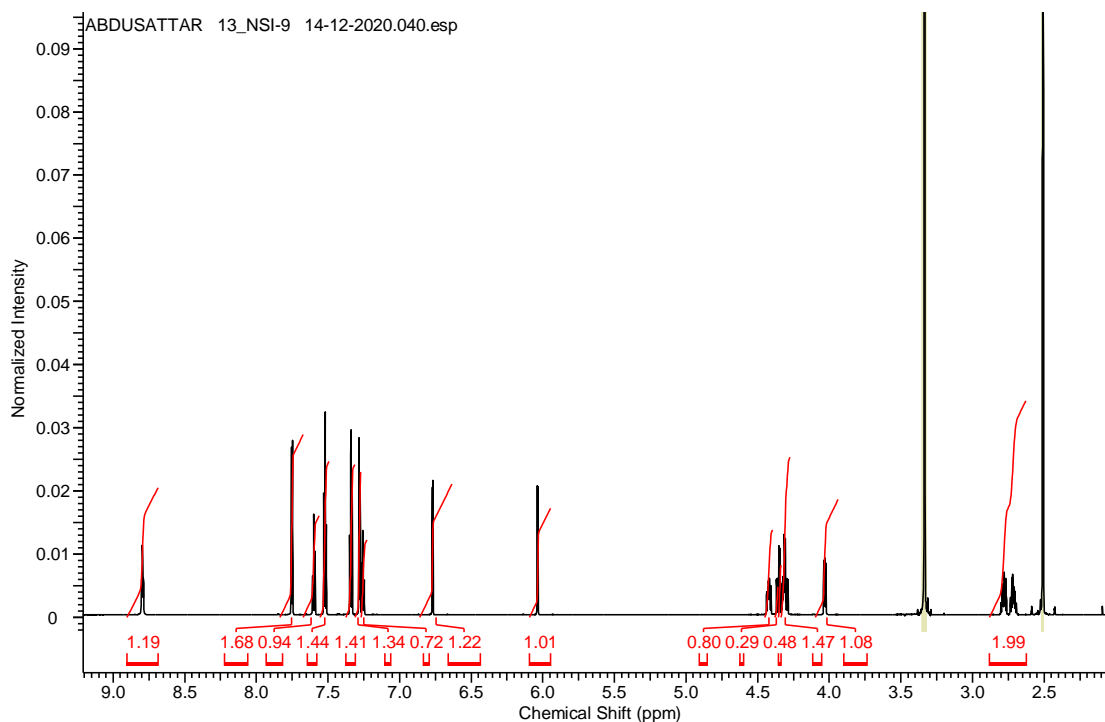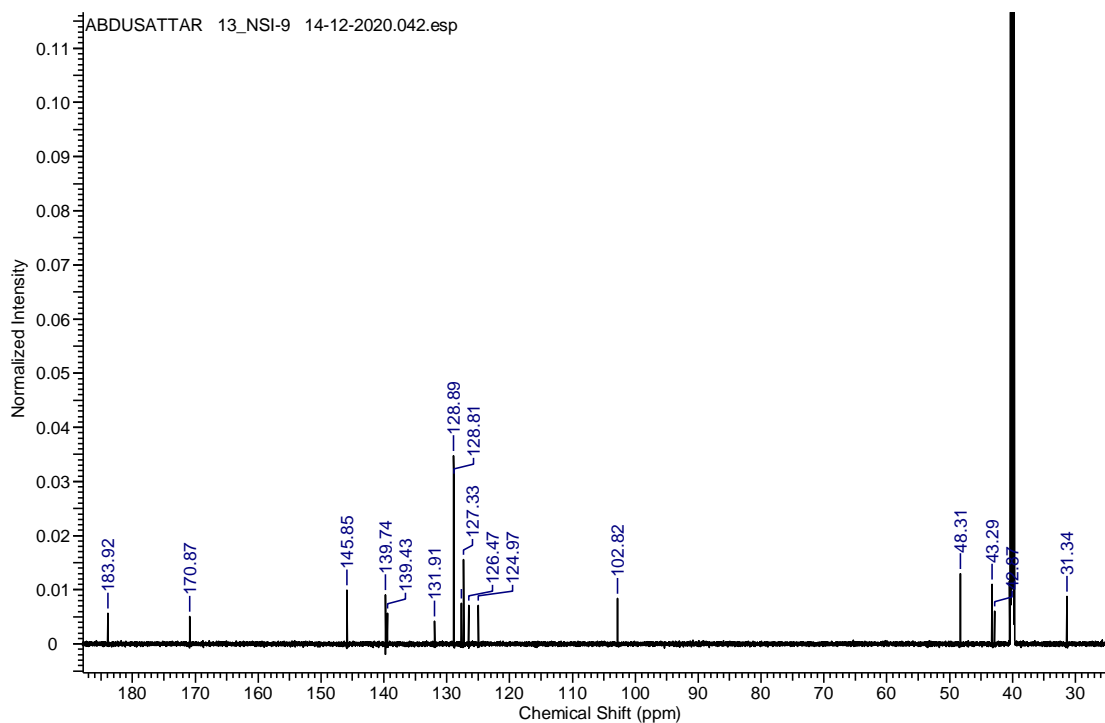

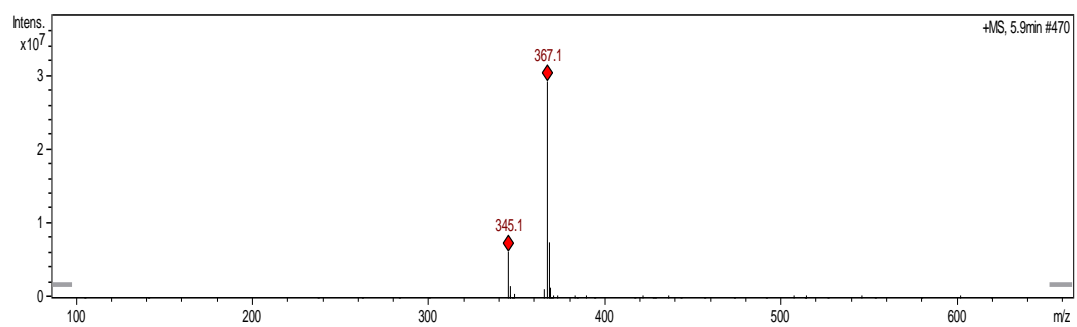

Supplement: Supplementary file 1 [file ijms-27-03850-s001.zip › ijms-4118644-supplementary.pdf]
